# Identification of lineage-associated polymorphisms in the ROP18 3′ flanking region and development of molecular assays for differentiation of *Toxoplasma gondii* lineages

**DOI:** 10.3389/fmicb.2026.1868079

**Published:** 2026-09-01

**Authors:** Bao-Yi Liang, Jia-Li Liu, Qiu-Mei Lin, Zhi-Hao Zhang, Rui-Ying Li, Jing-Hui Lu, Wei-Jie Chen, Zheng Jian, Fu-Shi Quan, Zhi-Ting Feng, Zhou-Rui Yin, Zhao-Shou Yang

**Affiliations:** 1 The First Affiliated Hospital/The First Clinical Medicine School of Guangdong Pharmaceutical University, Guangzhou, China; 2 Medical Research Center for Bioreaction to Reactive Oxygen Species and Biomedical Science Institute, Core Research Institute (CRI), Kyung Hee University, Seoul, Republic of Korea; 3 Department of Medical Zoology, School of Medicine, Kyung Hee University, Seoul, Republic of Korea; 4 Department of Pathology, the First Affiliated Hospital of Guangdong Pharmaceutical University, Guangzhou, China

**Keywords:** HRM-PCR, lineage-associated ROP18 patterns, molecular marker, ROP18 3′ flanking region, TaqMan MGB probe assay, *Toxoplasma gondii*

## Abstract

**Background:**

*Toxoplasma gondii* (*T. gondii*) exhibits substantial genetic diversity, and different parasite lineages are associated with distinct epidemiological distributions and biological characteristics. Accurate molecular characterization of *T. gondii* strains is important for understanding parasite population structure and transmission patterns. However, existing genotyping approaches often require multiple loci, extensive experimental procedures, or complex data analysis. Therefore, simplified and reliable molecular markers for rapid lineage differentiation are still needed.

**Methods:**

In this study, comparative genomic analysis was performed using representative *T. gondii* strains with well-defined genetic backgrounds and virulence phenotypes. The ROP18 genomic region, including partial genomic sequences, 5′ flanking regions, coding sequence (CDS), and 3′ flanking regions, was analyzed to identify informative polymorphic signatures. A short conserved sequence region containing lineage-associated polymorphic sites was identified within the ROP18 3′ flanking region. Based on these sequence signatures, HRM-PCR and TaqMan MGB probe-based real-time PCR assays were developed and evaluated using plasmid standards and representative *T. gondii* genomic DNA samples.

**Results:**

Phylogenetic analyses based on different ROP18 genomic regions demonstrated distinct clustering patterns among analyzed strains. Although the ROP18 3′ flanking region was highly conserved, a short conserved sequence region containing informative polymorphic sites was identified, and the combination of these sites generated three distinct lineage-associated ROP18 patterns. Analysis of publicly available genomic datasets further demonstrated that individual strains contained one of these defined patterns rather than multiple patterns simultaneously. The developed HRM-PCR assay successfully discriminated the three ROP18-associated patterns based on distinct melting profiles with good reproducibility. Furthermore, the TaqMan MGB probe-based assay enabled specific identification of different ROP18-associated patterns through defined probe-recognition combinations and showed good analytical performance.

**Conclusion:**

This study identifies novel lineage-associated molecular signatures within the ROP18 3′ flanking region and establishes complementary HRM-PCR and TaqMan MGB probe-based approaches for rapid molecular differentiation of *T. gondii* strains. These findings highlight the potential of conserved non-coding regions adjacent to functionally important genes as informative targets for parasite genotyping and provide a practical complementary tool for epidemiological surveillance and strain characterization.

## 1 Introduction

*Toxoplasma gondii* (*T. gondii*) is a globally distributed obligate intracellular protozoan parasite belonging to the phylum apicomplexa and is the causative agent of toxoplasmosis. Approximately one-third of the global population is estimated to be infected with *T. gondii*, and infection can result in severe clinical manifestations in immunocompromised individuals and congenitally infected fetuses, particularly involving neurological and ocular complications (Sengupta et al., 2025; Guan et al., 2023). *T. gondii* exhibits a complex life cycle, with felids serving as the definitive hosts and a wide range of warm-blooded animals, including humans, acting as intermediate hosts (Attias et al., 2020). The outcome of *T. gondii* infection is determined by a complex interplay among parasite genetic characteristics, parasite effector molecules, host immune responses, host genetic background, and environmental factors (Robert-Gangneux and Darde, 2012). The remarkable genetic diversity of *T. gondii* contributes to marked differences in virulence and geographic distribution, thereby influencing disease severity and epidemiological patterns (Brito et al., 2023). Therefore, accurate characterization of *T. gondii* lineages is essential for molecular epidemiological investigations, strain classification, and understanding parasite population structure.

The classical type I, type II, and type III lineages have historically been associated with distinct virulence phenotypes based on experimental mouse models using archetypal strains (Howe and Sibley, 1995; Su et al., 2002; Su et al., 2010; Sibley and Boothroyd, 1992). Under these experimental conditions, type I strains generally exhibit high virulence, whereas type II and type III strains typically show intermediate and low virulence phenotypes, respectively (Su et al., 2002; Sibley and Boothroyd, 1992). However, these observations are derived from representative classical strains and should not be generalized to all isolates, particularly atypical and recombinant genotypes that exhibit substantial genetic diversity and variable biological characteristics (Su et al., 2010; Khan et al., 2007; Lehmann et al., 2006). Phylogenetic analyses based on intron and multilocus sequence variation have revealed extensive genetic diversity and geographic structuring of *T. gondii* populations, including multiple haplogroups with distinct evolutionary histories across North America, Europe, and South America (Khan et al., 2007; Khan et al., 2005).

Several molecular approaches have been developed for the genetic characterization and lineage identification of *T. gondii*. Conventional multilocus genotyping methods, including PCR-RFLP, microsatellite analysis (MS), multilocus sequence typing (MLST), RAPD, and intron sequence analysis, have greatly contributed to understanding the population structure and evolutionary history of *T. gondii* (Su et al., 2006; Ajzenberg et al., 2010; Su et al., 2012; Koutsogiannis and Denny, 2024; Khan et al., 2011; Liu et al., 2015; Rahdar et al., 2021; Jokelainen et al., 2017; Azimpour-Ardakan et al., 2021; Lorenzi et al., 2016). These approaches have enabled the differentiation of classical clonal lineages and the identification of atypical or recombinant genotypes. However, many of these methods require analysis of multiple genetic loci, extensive experimental optimization, specialized technical expertise, or complex data interpretation, which may limit their application in rapid and routine screening of large numbers of samples.

Among the genomic regions explored for *T. gondii* strain differentiation, rhoptry protein genes have attracted considerable attention due to their extensive genetic diversity and biological relevance during host–parasite interactions. ROP18, encoding a polymorphic serine/threonine kinase secreted from the rhoptry organelles, is one of the best-characterized *T. gondii* effector molecules involved in host immune modulation (Saeij et al., 2006; Taylor et al., 2006). Previous studies have demonstrated that ROP18 contributes to parasite survival by phosphorylating host immunity-related GTPases and interfering with IFN-*γ*-mediated host defense mechanisms, thereby influencing parasite fitness and virulence phenotypes in experimental models (Saeij et al., 2006; Fentress et al., 2010).

ROP18 is encoded by a single-copy gene, and the ROP18 locus exhibits substantial sequence diversity among different *T. gondii* lineages, making it an attractive target for molecular typing and evolutionary studies (Khan et al., 2009; Costa et al., 2025; Shwab et al., 2016; Fukumoto et al., 2020). However, sequence polymorphisms identified outside the ROP18 coding region, including those in the flanking region, should be considered as lineage-associated molecular signatures rather than direct indicators of parasite virulence. Previous studies have primarily focused on polymorphisms within the ROP18 coding region or regulatory sequences located in the 5′ flanking region, particularly the upstream segment (UPS), to investigate lineage-specific variation and virulence-associated phenotypes (Khan et al., 2009; Subekti et al., 2021). The potential utility of flanking regions surrounding the ROP18 locus, especially the 3′ flanking region, as molecular markers for *T. gondii* classification has not been systematically explored. Therefore, this study aimed to identify lineage-associated polymorphic signatures within the ROP18 3′ flanking region and develop molecular assays for differentiation of classical T. gondii lineages.

## 2 Materials and methods

### 2.1 Data sources and genome retrieval

Complete genome sequences and corresponding annotations for 18 representative *T. gondii* strains were initially retrieved from ToxoDB (release 46, 06 November 2019).^1^ The classical lineage, reported virulence phenotype, strain information, and ROP18 allele type of the 18 *T. gondii* strains are summarized in Table 1 (Khan et al., 2009; Clawson et al., 2023; Zhang et al., 2019).

**Table 1:** The classical lineage, reported virulence phenotype, and ROP18 allele type of the 18 *T. gondii* strains.

Strain name	Classical lineage	Virulence	ROP18 allele type*	Strain name	Classical lineage	Virulence	ROP18 allele type*
CAST	I	Highly virulent	I	ME49	II	Intermediate virulence	II
CtCo5	I	Highly virulent	I	TgCatPRC2	II	Intermediate virulence	–
FOU	I	Highly virulent	I	p89	III	Intermediate virulence	III
GT1	I	Highly virulent	I	VEG	III	Avirulent	III
RH 2016	I	Highly virulent	–	MAS	Atypical	Highly virulent	I
RH-88	I	Highly virulent	–	RUB	Atypical	Highly virulent	I
TgCatBr9	I	Highly virulent	I	TgCatBr5	Atypical	Highly virulent	–
ARI	II	Intermediate virulence	–	VAND	Atypical	Highly virulent	I
COUG	II	Intermediate virulence	–	GAB2-2007-GAL-DOM2	Atypical	–	–

“–” indicates that the information has not been reported in publicly available literature. “*”: The ROP18 allele type was assigned according to the classification reported by Khan et al. (2009).

The ROP18 locus was identified based on available ROP18 gene/protein annotations in the corresponding genome assemblies. For genome datasets with annotated ROP18 proteins, the corresponding genomic coordinates (chromosome location, start position, end position, and strand information) were directly obtained from the database annotation. For genomes lacking ROP18 annotations, the ROP18 reference sequence from the ME49 genome was used as a query for BLAST searching against the corresponding genome assembly, and the genomic coordinates of the best matching region were determined for subsequent sequence extraction. The corresponding ROP18 protein sequences were deduced from the coding sequences using DNAMAN software (Lynnon Biosoft, Canada) and subsequently used for multiple sequence alignment and phylogenetic analysis.

The genomic regions surrounding the ROP18 coding sequence were subsequently extracted according to the identified coordinates. For ROP18 loci located on the reverse strand, the corresponding genomic region was selected according to the annotated strand information in the genome browser, and the reverse-strand option was applied during sequence export to obtain FASTA sequences in the correct transcriptional orientation. All extracted sequences were exported in FASTA format for subsequent multiple sequence alignment and phylogenetic analyses. The extracted ROP18 genomic regions, genomic coordinates, strand orientations, and corresponding ROP18 protein information are summarized in Table 2.

**Table 2:** Genomic and protein annotations of the ROP18 locus in *T. gondii* strains from ToxoDB.

Strain name	Extracted partial genomic sequences (5′–3′)					ROP18 protein	
	Accession No. and Region (length)	Strand	5′-Flank region (2,000 bp)	CDS region (length)	3′-Flank region (2,000 bp)	Accession No.	Length (AA)
CAST	ML130720:3093978-3099642 (5,665 bp)	+	ML130720:3093978-3095977	ML130720:3095978-3097642 (1,665 bp)	ML130720:3097643-3099642	TGCAST_205250	554
CtCo5	AKIR01000101:c43187-37523 (5,665 bp)	−	AKIR01000101:c43187-41188	AKIR01000101:c41187-39523 (1,665 bp)^**a**^	AKIR01000101:c39522-37523	NA^b^	554
FOU	KN000265:c363782-358118 (5,665 bp)	−	KN000265:c363782-361783	KN000265:c361782-360118 (1,665 bp)	KN000265:c360117-358118	TGFOU_205250	554
GT1	TGGT1_chrVIIa:c1430446-1424782 (5,665 bp)	−	TGGT1_chrVIIa:c1430446-1428447	TGGT1_chrVIIa:c1428446-1426782 (1,665 bp)	TGGT1_chrVIIa:c1426781-1424782	TGGT1_205250	554
MAS	KN044642:c367472-361808 (5,665 bp)	−	KN044642:c367472-365473	KN044642:c365472-363808 (1,665 bp)	KN044642:c363807-361808	TGMAS_205250	554
RH 2016	LLKL01000076:198975-204639 (5,665 bp)	+	LLKL01000076:198975-200974	LLKL01000076:200975-202639 (1,665 bp)^a^	LLKL01000076:202640-204639	NA^b^	554
RH-88	CM023088:c1599550-1593886 (5,665 bp)	−	CM023088:c1599550-1597551	CM023088:c1597550-1595886 (1,665 bp)	CM023088:c1595885-1593886	TGRH88_034260	554
RUB	KN040001:191277-196941 (5,665 bp)	+	KN040001:191277-193276	KN040001:193277-194941 (1,665 bp)	KN040001:194942-196941	TGRUB_205250	554
TgCatBr5	AFPV01003518:17723-23387 (5,665 bp)	+	AFPV01003518:17723-19722	AFPV01003518:19723-21387 (1,665 bp)^**a**^	AFPV01003518:21388-23387	NA^b^	554
TgCatBr9	KZ791615:c1021405-1015741 (5,665 bp)	−	KZ791615:c1021405-1019406	KZ791615:c1019405-1017741 (1,665 bp)	KZ791615:c1017740-1015741	TGBR9_205250	554
VAND	KN042496:c364389-358725 (5,665 bp)	−	KN042496:c364389-362390	KN042496:c362389-360725 (1,665 bp)	KN042496:c360724-358725	TGVAND_205250	554
ARI	KQ973610:c1577901-1572237 (5,665 bp)	−	KQ973610:c1577901-1575902	KQ973610:c1575901-1574237 (1,665 bp)	KQ973610:c1574236-1572237	TGARI_205250	554
COUG	KZ624886:289771-295435 (5,665 bp)	+	KZ624886:289771-291770	KZ624886:291771-293435 (1,665 bp)	KZ624886:293436-295435	TGCOUG_205250	554
ME49	NC_031474:c1518113-1512449 (5,665 bp)	−	NC_031474:c1518113-1516114	NC_031474:c1516113-1514449 (1,665 bp)	NC_031474:c1514448-1512449	TGME49_205250	554
TgCatPRC2	KQ983279:1085304-1090968 (5,665 bp)	+	KQ983279:1085304-1087303	KQ983279:1087304-1088968 (1,665 bp)	KQ983279:1088969-1090968	TGPRC2_205250	554
p89	KL998096:c1034257-1028587 (5,671 bp)	−	KL998096:c1034257-1032258	KL998096:c1032257-1030587 (1,671 bp)	KL998096:c1030586-1028587	TGP89_205250	556
VEG	TGVEG_chrVIIa:c1588482-1582812 (5,671 bp)	−	TGVEG_chrVIIa:c1588482-1586483	TGVEG_chrVIIa:c1586482-1584812 (1,671 bp)	TGVEG_chrVIIa:c1584811-1582812	TGVEG_205250	556
GAB2-2007-GAL-DOM2	KN003127:c373697-368027 (5,671 bp)	−	KN003127:c373697-371788	KN003127:c371697-370027 (1,671 bp)	KN003127:c370028-368027	TGDOM2_205250	556

“a” indicates that the corresponding sequences were derived from the extracted partial genome sequences; “b” indicates that the ROP18 protein sequence was unavailable from the ToxoDB database and was predicted based on the corresponding ROP18 nucleotide sequence.

The partial ROP18-associated genomic sequences of three representative strains, RH (GenBank accession no. OQ376745), Pru (OQ376746), and CTG (OQ376747), were generated and deposited by our research group in the NCBI GenBank database. Publicly available raw sequencing reads of genomes were obtained from the NCBI Sequence Read Archive (SRA) database.^2^ Raw sequencing reads reported in previous studies, including datasets described by Lorenzi et al. (2016), were retrieved according to the corresponding SRA accession numbers. In addition, SRA experiment datasets associated with BioProject accession no. PRJDB16997, which were generated and deposited by previous researchers, were retrieved from the public database and reused in the present study for targeted recovery and comparative analysis of ROP18-associated genomic regions.

### 2.2 Phylogenetic analysis

Phylogenetic analyses were performed based on previously published phylogenetic analysis strategies for *T. gondii* (Khan et al., 2009). Phylogenetic trees were constructed using MEGA12 software.^3^ Multiple sequence alignments of nucleotide and amino acid sequences were initially performed using the ClustalW algorithm implemented in MEGA12. The aligned nucleotide and protein sequences were subsequently used for phylogenetic reconstruction.

Neighbor-joining (NJ) trees were constructed with 1,000 bootstrap replicates to evaluate the reliability of the inferred evolutionary relationships. For nucleotide sequence analysis, evolutionary distances were calculated using the Maximum Composite Likelihood method. For amino acid sequence analysis, evolutionary distances were estimated using the Poisson correction model. Gaps and missing data were treated using the pairwise deletion option. Bootstrap values were calculated and displayed on the corresponding branches. The phylogenetic trees generated by MEGA12 were presented in an unrooted topology, exported, and further edited using WPS Office for visualization and figure preparation.

### 2.3 Multiple sequence alignment and identification of lineage-associated polymorphic patterns

Multiple sequence alignments of the extracted *Toxoplasma gondii* ROP18 genomic regions were performed using DNAMAN software (Lynnon Biosoft, Canada). The full alignment option was selected for sequence alignment. Pairwise alignment was performed using the Quick Alignment algorithm with a gap penalty of 7, a K-tuple value of 2, a window size of 4, and four top diagonals. Multiple sequence alignment was performed using the following parameters: gap opening penalty of 10, gap extension penalty of 5, DNA transition weight of 0.5, DNA weight matrix of IUB, protein weight matrix of GONNET, and delay divergent cutoff of 30. Hydrophilic penalties, residue-specific penalties, and gap separation distance were set according to the default parameters of DNAMAN software.

The aligned sequences were visually inspected to identify conserved regions within the ROP18 genomic region. Candidate short conserved sequence regions were selected based on the following criteria: (i) the region was highly conserved among analyzed strains with limited background sequence variation; (ii) the region contained informative polymorphic sites, including nucleotide substitutions or insertion/deletion variations; and (iii) the combination of polymorphic sites generated distinct sequence signatures capable of discriminating strains belonging to different lineage-associated groups. The selected region was subsequently defined as the lineage-associated ROP18 pattern region and was further used for molecular assay development.

### 2.4 Identification of lineage-associated ROP18 patterns from SRA datasets

To determine the distribution of lineage-associated ROP18 patterns in publicly available *T. gondii* genomic datasets, the identified pattern sequences were used as queries for screening SRA datasets. Three representative ROP18 pattern sequences were selected as reference markers, including the Type I-associated pattern from the RH genome (GenBank accession no. OQ376745), the Type II-associated pattern from the Pru genome (GenBank accession no. OQ376746), and the Type III-associated pattern from the CTG genome (GenBank accession no. OQ376747).

The representative pattern sequences (~96–97 bp) were used as query sequences for BLASTN searches against the NCBI Sequence Read Archive (SRA) database. Searches were performed using the megablast algorithm, which is optimized for highly similar nucleotide sequences. The E-value threshold was set at 10, and up to 100 significant matches were retrieved. Hits with query coverage ≥95% and nucleotide identity ≥98% were considered positive matches. SRA datasets containing reads corresponding to one of the three predefined lineage-associated ROP18 patterns were selected for subsequent targeted sequence recovery and pattern assignment analysis.

For targeted recovery of the ROP18-associated region from selected SRA datasets, representative reference genomic sequences from RH, Pru, and CTG were used. Because raw SRA reads are generally short and may not span the entire target region, the reference sequences containing the ROP18 locus were divided into overlapping fragments and used as queries for BLASTN screening against the corresponding SRA datasets. Reads matching the target fragments were retrieved according to the following criteria: sequence identity ≥98%, query coverage ≥90%, and E-value ≤1 × 10^−5^.

Retrieved reads were imported into DNAMAN software for sequence assembly based on overlapping regions. The recovered sequences were required to span the target ROP18-associated region without unresolved gaps to allow identification of the informative polymorphic sites. Samples lacking complete information for the four informative polymorphic sites were excluded from downstream ROP18 pattern assignment.

### 2.5 Genomic DNA extraction

*Toxoplasma gondii* strains were propagated in human foreskin fibroblast (HFF) cell cultures under standard culture conditions. When intracellular tachyzoites were fully replicated and the majority of infected HFF cells were undergoing parasite-induced lysis, all cells were collected for genomic DNA extraction.

Genomic DNA was extracted using the TIANamp Genomic DNA Kit (Tiangen Biotech, Beijing, China) according to the manufacturer’s instructions. During parasite collection from HFF cultures, a small amount of residual host-cell genomic DNA may have been co-extracted with parasite genomic DNA. Therefore, the extracted DNA samples represented a biologically relevant mixed DNA background containing predominantly *T. gondii* genomic DNA with trace amounts of host genomic DNA.

The concentration and purity of extracted genomic DNA were assessed using a NanoDrop spectrophotometer (Thermo Fisher Scientific, USA). DNA purity was evaluated based on the absorbance ratios at 260/280 nm and 260/230 nm. DNA samples with an A260/A280 ratio of approximately 1.8–2.0 and an A260/A230 ratio above 1.8 were considered suitable for downstream PCR analysis. The extracted genomic DNA was stored at −20 °C until use.

Genomic DNA extracted from THP-1 human monocytic cells and RAW264.7 mouse macrophage cells was used as host genomic DNA controls. Briefly, cells were collected by centrifugation and washed with phosphate-buffered saline (PBS). Genomic DNA was extracted using a commercial genomic DNA extraction kit (TIANGEN Biotech, Beijing, China) according to the manufacturer’s instructions. The concentration and purity of extracted DNA were determined using a NanoDrop spectrophotometer by measuring the absorbance ratios at 260/280 nm. The extracted genomic DNA samples were stored at −20 °C until further analysis.

### 2.6 Construction and synthesis of plasmid standards

To validate primer/probe specificity and provide standardized positive control templates for PCR-based assays, plasmid standards containing representative *T. gondii* ROP18 3′ flanking region sequences were synthesized by TsingKe Biological Technology Co., Ltd. (Beijing, China). Three plasmid standards were generated by cloning the selected ROP18 3′ flanking fragments representing the Type I-, Type II-, and Type III-associated ROP18 patterns into the provided plasmid vectors. Each inserted fragment contained the corresponding informative polymorphic sites used for lineage-associated pattern differentiation.

**Figure 1:**
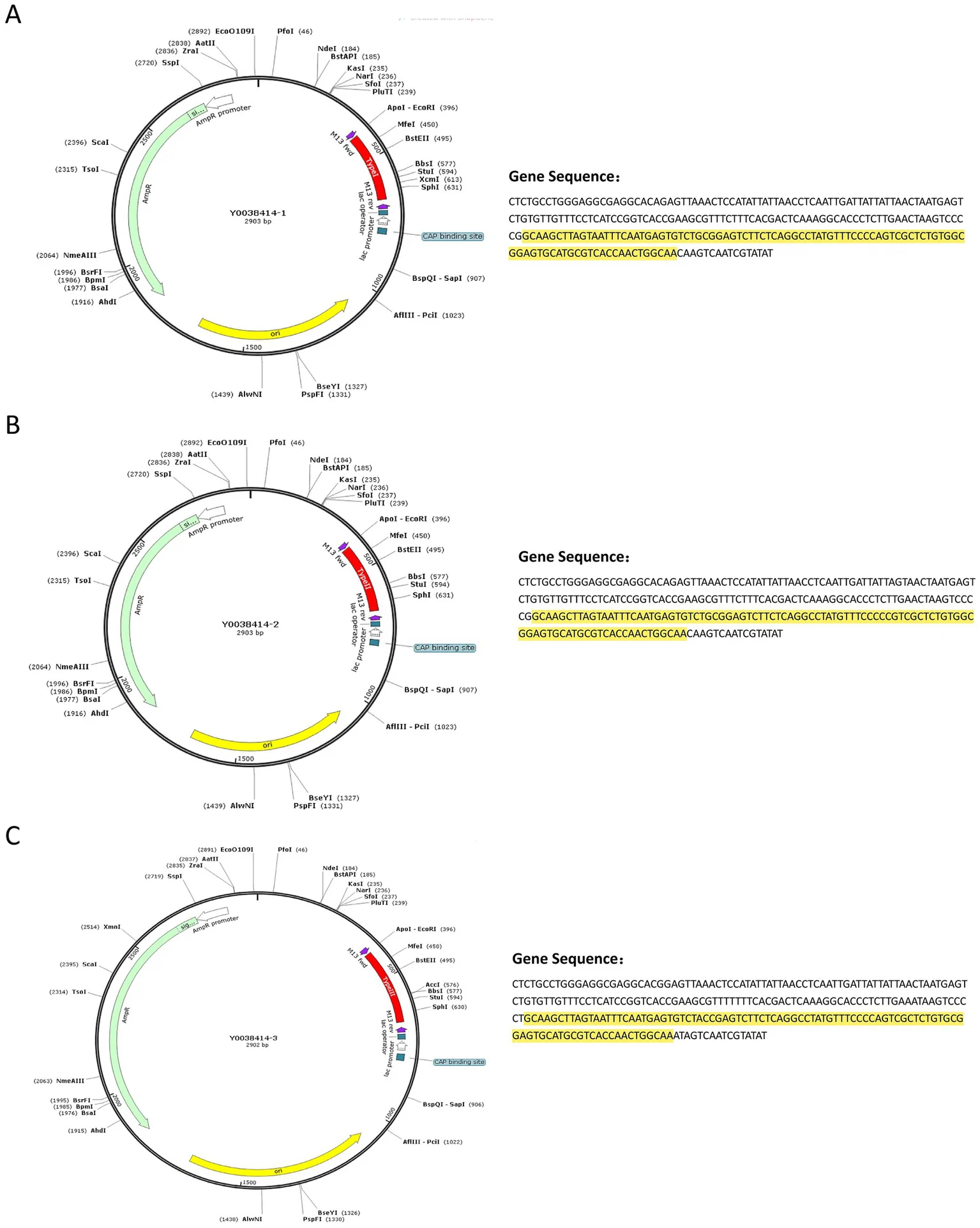
Construction and characterization of plasmid standards containing lineage-associated ROP18 sequence patterns. Plasmids containing the representative ROP18 lineage-associated sequence patterns were synthesized and used as genotype-specific standards for HRM-PCR and TaqMan probe-based real-time PCR assays. **(A–C)** Circular Maps of plasmid standards containing Type I, Type II, and Type III ROP18 lineage-associated sequences, respectively. The inserted target sequences are highlighted in yellow.

The recombinant plasmids were purified using an endotoxin-free plasmid extraction procedure performed by the company. Plasmid DNA concentration was determined, and the copy number of each plasmid standard was calculated based on plasmid molecular weight and DNA concentration using an established online calculation formula (Kuhar et al., 2013). The plasmid standards were subsequently serially diluted to generate templates for analytical sensitivity evaluation, amplification efficiency determination, and intra−/inter-assay reproducibility assessment of the HRM-PCR and TaqMan probe-based assays. The schematic maps of the plasmid constructs and the corresponding inserted sequences are shown in Figure 1.

### 2.7 Primer and TaqMan MGB probe design

Primers and TaqMan MGB probes were designed based on the multiple sequence alignment results of representative *T. gondii* strains carrying different lineage-associated ROP18 patterns. The conserved regions surrounding the informative polymorphic sites within the ROP18 3′ flanking region were identified and selected as the target region for assay development. Candidate primer sequences were designed using the NCBI Primer-BLAST online tool.^4^ The locations of the identified lineage-associated ROP18 patterns, primer binding sites, probe target regions, and informative polymorphic sites were mapped and visualized using SnapGene software.^5^

Primer selection was performed according to the following criteria: (i) primers were located within conserved regions to ensure consistent amplification among different *T. gondii* strains; (ii) the amplified fragment contained informative polymorphic sites suitable for lineage-associated pattern differentiation; (iii) primer length, melting temperature (Tm), and GC content were within the recommended ranges for PCR amplification; (iv) potential secondary structures, including hairpin structures and primer-dimer formation, were minimized; and (v) primer specificity was confirmed by BLAST analysis against non-target sequences. Candidate primer pairs were experimentally evaluated by PCR amplification, and the final primer pair was selected based on the generation of a single specific amplicon with the expected size, efficient amplification, and good reproducibility.

The selected primer pair was: Forward primer: 5′-GCAAGCTTAGTAATTTCAATGAGT-3′; Reverse primer: 5′-TTGCCAGTTGGTGACGCATG-3′. The final primer pair amplified the ROP18 3′ flanking region containing the lineage-associated polymorphic sites, generating amplicons of approximately 96–97 bp for subsequent HRM-PCR and TaqMan MGB probe-based assays.

For TaqMan MGB probe design, probe sequences were selected based on the lineage-associated polymorphic sites identified within the ROP18 3′ flanking region. Candidate probe sequences were designed according to established criteria. The probe design criteria included: (i) the probe was located within the amplified region and covered the target polymorphic site; (ii) the informative polymorphic nucleotide was positioned near the central region of the probe to enhance allele-specific differentiation; (iii) the probe sequence showed high specificity toward the target *T. gondii* sequence without significant homology to non-target sequences; (iv) probe length, GC content, and melting temperature (Tm) were optimized to ensure efficient hybridization; and (v) potential secondary structures, including hairpin formation and probe-dimer formation, were avoided.

Three MGB probes were subsequently designed to recognize different lineage-associated ROP18 sequence patterns through combined fluorescence profiles (Kutyavin et al., 2000). The probe sequences were as follows: Probe 1: 5′-FAM-AAGACTCCGCAGACAC-MGB-3′; Probe 2: 5′-VIC-AAGACTCGGTAGAACAC-MGB-3′; Probe 3: 5′-ROX-TTTCCCCAGTCGCTCT-MGB-3′. The probe combinations and expected fluorescence profiles for representative Type I (RH), Type II (Pru), and Type III (CTG) pattern sequences were predefined and are summarized in Table 3.

**Table 3:** Sequences and genomic positions of TaqMan MGB probes targeting lineage-associated ROP18 patterns.

Probe	Fluorescent dye	Target lineage-associated ROP18 pattern	Reference strain	GenBank accession	Target nucleotide position
Probe 1	FAM	Type I/Type II-associated ROP18 pattern	RH/Pru	OQ376745/OQ376746	c3134–3149
Probe 2	VIC	Type III-associated ROP18 pattern	CTG	OQ376747	c3139–3154
Probe 3	ROX	Type I/Type III-associated ROP18 pattern	RH/CTG	OQ376745/OQ376747	c3162–3177 (RH); c3167–3182 (CTG)

Probe 1 and Probe 3 recognize the shared patterns between Type I and Type II or Type I and Type III lineages, respectively, whereas Probe 2 specifically recognizes the Type III-associated pattern. The fluorescent dyes FAM, VIC, and ROX were used for detection of Probe 1, Probe 2, and Probe 3, respectively.

### 2.8 Optimization of real-time PCR conditions

The real-time PCR conditions were optimized to achieve efficient and specific amplification of the target ROP18 flanking region. Different primer and probe concentrations and annealing temperatures were evaluated to determine the optimal reaction conditions. The optimal reaction conditions were selected according to the following criteria: (i) strong and reproducible amplification of the target sequence; (ii) low variation in Ct values among technical replicates; (iii) absence of non-specific amplification or primer/probe-dimer signals; and (iv) high amplification efficiency with good linearity across template concentrations. The optimized parameters were subsequently applied for all real-time PCR assays.

### 2.9 HRM-PCR assay

High-resolution melting (HRM) analysis was performed to identify sequence variations within the ROP18 3′ flanking region using an HRM analysis kit containing EvaGreen fluorescent dye (FP210, TIANGEN Biotech Co., Ltd., Beijing, China) on a LightCycler® 480 Real-Time PCR System (Roche Diagnostics, Mannheim, Germany).

The HRM-PCR reaction was performed in a final volume of 20 μL containing HRM master mix, ROP18-specific forward and reverse primers, and template DNA. The final concentrations of both forward and reverse primers were 0.3 μM. PCR amplification was conducted with an initial denaturation step at 95 °C for 5 min, followed by 45 cycles of 95 °C for 15 s and 63 °C for 1 min. Fluorescence signals were acquired at the end of each annealing/extension step using the 465 nm excitation and 510 nm emission detection channel. Following amplification, HRM analysis was performed by gradually increasing the temperature from 65 °C to 95 °C at a ramp rate of 0.02 °C/s, with continuous fluorescence acquisition during the melting phase. HRM data were analyzed using the LightCycler® 480 Gene Scanning Software.

For melting curve analysis, fluorescence data were normalized using the pre- and post-melting regions. The normalization regions were manually adjusted based on the melting behavior of the amplified products, with the pre-melting region set from 78.31 °C to 79.18 °C and the post-melting region set from 82.98 °C to 83.88 °C. Normalized melting curves were generated, and difference plots were constructed to visualize sequence-dependent variations among samples. The clustering sensitivity parameter in the Gene Scanning analysis was set to 0.39 to optimize differentiation among different melting profiles.

### 2.10 HRM-PCR pattern assignment criteria

HRM-PCR pattern assignment was performed by comparison with reference melting profiles generated from plasmid standards and representative genomic DNA controls of RH, Pru, and CTG strains, representing Type I-, Type II-, and Type III-associated ROP18 patterns, respectively. Classification was based on the concordant clustering of samples with the corresponding reference profiles according to multiple HRM parameters, including normalized melting curves, melting peak profiles, and temperature-shifted difference plots.

Samples were assigned to a specific lineage-associated ROP18 pattern only when their melting profiles consistently matched the corresponding reference control across replicate experiments. Classification was not determined solely based on melting temperature (Tm) values, but rather on the overall melting behavior and clustering pattern relative to the reference controls.

Samples showing intermediate melting profiles, inconsistent clustering among replicates, multiple melting peaks, abnormal melting behavior, or profiles lacking correspondence with any predefined reference pattern were considered indeterminate and were not assigned to a specific lineage-associated ROP18 pattern.

### 2.11 TaqMan probe-based real-time PCR assay

A TaqMan MGB probe-based real-time PCR assay was developed for differentiation of lineage-associated ROP18 patterns based on informative polymorphic sites within the ROP18 3′ flanking region. The primer and probe sequences were designed according to the multiple sequence alignment results of representative *T. gondii* strains. The polymorphic nucleotides associated with different ROP18 patterns were located within the probe-binding regions to enable sequence-dependent fluorescence differentiation. During the development of the TaqMan assay, multiplex probe detection was initially evaluated. However, fluorescence signal interference and overlap among different probes were observed, which affected accurate allele differentiation. Therefore, a singleplex TaqMan real-time PCR format was selected for subsequent experiments to achieve clearer fluorescence separation and reliable genotype identification.

Real-time PCR reactions were performed using the Taq Pro Universal Probe qPCR Master Mix (AG11730; Accurate Biology, Changsha, China) on a qTOWER^3^/G Real-Time PCR System (Analytik Jena AG, Germany). Each reaction was performed in a final volume of 20 μL, containing qPCR master mix, ROP18-specific forward and reverse primers, corresponding fluorescent TaqMan MGB probes, and template DNA. The final concentrations of both forward and reverse primers were 0.3 μM. The concentrations of the three probes were optimized as follows: Probe 1 (FAM-labeled), 0.1 μM; Probe 2 (VIC-labeled), 0.2 μM; and Probe 3 (ROX-labeled), 0.1 μM.

Amplification was performed using the following thermal cycling conditions: initial denaturation at 95 °C for 5 min, followed by 45 cycles of 95 °C for 15 s and 63 °C for 1 min. Fluorescence signals were collected at the end of each annealing/extension step during PCR amplification. The excitation/emission settings and fluorescence acquisition parameters were optimized according to the detection channels of the qTOWER^3^/G system. The FAM channel was detected at 470/520 nm with a gain setting of 3, the VIC channel at 515/545 nm with a gain setting of 8, and the ROX channel at 535/580 nm with a gain setting of 10. The fluorescence threshold settings were set as 8 for FAM and VIC channels and 10.5 for the ROX channel.

Recombinant plasmids containing representative Type I-, Type II-, and Type III-associated ROP18 pattern sequences were used as positive controls to establish reference fluorescence profiles. Negative controls containing nuclease-free water instead of template DNA were included in each experiment to monitor potential contamination. Genotype assignment was performed according to the combined fluorescence profiles generated by the three TaqMan MGB probes, with each ROP18 pattern defined by its specific probe recognition profile.

### 2.12 TaqMan pattern assignment criteria

Each sample was analyzed using three independent singleplex TaqMan reactions, and pattern assignment was performed based on the combined fluorescence profiles of Probe 1, Probe 2, and Probe 3. Based on the identified lineage-associated ROP18 polymorphic patterns, a predefined probe recognition matrix was established (Table 4). These probe recognition profiles were established using representative plasmid standards and genomic DNA controls from RH, Pru, and CTG strains, respectively.

**Table 4:** Probe recognition matrix for lineage-associated ROP18 pattern assignment.

Lineage-associated ROP18 pattern	Probe 1 (FAM)	Probe 2 (VIC)	Probe 3 (ROX)	Classification profile
Type I-associated pattern	+	−	+	Probe 1^+^/Probe 2^−^/Probe 3^+^
Type II-associated pattern	+	−	−	Probe 1^+^/Probe 2^−^/Probe 3^−^
Type III-associated pattern	−	+	+	Probe 1^−^/Probe 2^+^/Probe 3^+^

A positive reaction was defined as a reproducible amplification curve crossing the preset fluorescence threshold with a Ct value within the acceptable analytical range. Amplification signals detected only at late cycles (Ct > 35), inconsistent amplification among replicate reactions, or signals lacking reproducibility were considered uncertain and required repeat testing. Negative reactions were defined as the absence of detectable amplification under the established assay conditions.

Samples were assigned to a lineage-associated ROP18 pattern only when the complete Probe 1/Probe 2/Probe 3 recognition profile was concordant with one of the predefined classification matrices. Samples showing discordant replicate results, unexpected probe combinations, absence of amplification in all three reactions, or profiles inconsistent with the three reference patterns were classified as indeterminate.

### 2.13 Analytical specificity

The analytical specificity of the HRM-PCR and TaqMan MGB probe-based real-time PCR assays was evaluated using both *Toxoplasma gondii* plasmid standards and mammalian genomic DNA. Human THP-1 cell genomic DNA and mouse RAW264.7 cell genomic DNA were used as non-target DNA controls to assess potential non-specific amplification from host genomic backgrounds.

### 2.14 Analytical sensitivity and assay reproducibility

The analytical sensitivity of the HRM-PCR and TaqMan MGB probe-based real-time PCR assays was evaluated using serially diluted recombinant plasmid standards with known copy numbers. A series of 10-fold serial dilutions covering a broad range of template concentrations was prepared and analyzed to assess the amplification performance and detection capability of the established assays. Analytical sensitivity was evaluated using serially diluted recombinant plasmid standards ranging from 1 × 10^3^ to 1 × 10^7^ copies/reaction. The lowest tested concentration generating reproducible amplification signals was recorded, and standard curves were generated to evaluate linearity and amplification efficiency.

Amplification efficiency was calculated from the slope of the standard curve according to the equation:


$$E=(10^{-1}{/}^{\text{slope}}-1)\times 100\%$$

For the HRM-PCR assay, analytical sensitivity was evaluated based on the lowest plasmid concentration that generated a detectable amplification product and produced a reproducible melting profile corresponding to the expected ROP18 3′ flanking region pattern. For the TaqMan MGB probe-based real-time PCR assay, analytical sensitivity was evaluated separately for each probe by analyzing amplification curves and fluorescence signals generated across the tested plasmid concentration range.

Standard curves were generated by plotting the threshold cycle (Ct) values against the logarithm of plasmid copy numbers. The amplification efficiency and linearity of each TaqMan assay were calculated based on the slope and correlation coefficient (*R*^2^) of the standard curves.

The intra-assay and inter-assay reproducibility of the established HRM-PCR and TaqMan MGB probe-based real-time PCR assays were evaluated using recombinant plasmid standards containing representative ROP18 pattern sequences. For intra-assay variability analysis, selected plasmid concentrations within the tested range were analyzed in triplicate within the same PCR run. For the TaqMan assay, the mean Ct values, standard deviations (SD), and coefficients of variation (CV%) were calculated. For the HRM-PCR assay, reproducibility was assessed by comparing melting curve profiles and pattern clustering results among replicate reactions.

For inter-assay variability analysis, the same plasmid standards were tested in independent experiments performed on different days. The mean Ct values, SD, and CV% were calculated for the TaqMan assay, while the consistency of melting profiles and pattern assignment was evaluated for the HRM-PCR assay. Assay reproducibility was determined based on the consistency of amplification performance, fluorescence signal generation, and pattern classification across repeated experiments.

### 2.15 Agarose gel electrophoresis

Agarose gel electrophoresis was performed for analysis of PCR-amplified DNA fragments. Briefly, PCR products were mixed with 6 × DNA loading dye (AG11902, Accurate Biology, China) and loaded onto 1.2% agarose gels prepared in 1 × TAE buffer. A GL DNA Marker 500 (AG11905, Accurate Biology, China) was loaded alongside the samples as a molecular weight marker. Electrophoresis was carried out at a constant voltage of 120 V for 20 min. Following electrophoresis, DNA bands were visualized using a Bio-Rad gel documentation system (Bio-Rad Laboratories, Hercules, CA, USA), and images were captured for subsequent analysis.

## 3 Results

### 3.1 Identification of lineage-associated polymorphic signatures within the ROP18 3′ flanking region

**Figure 2:**
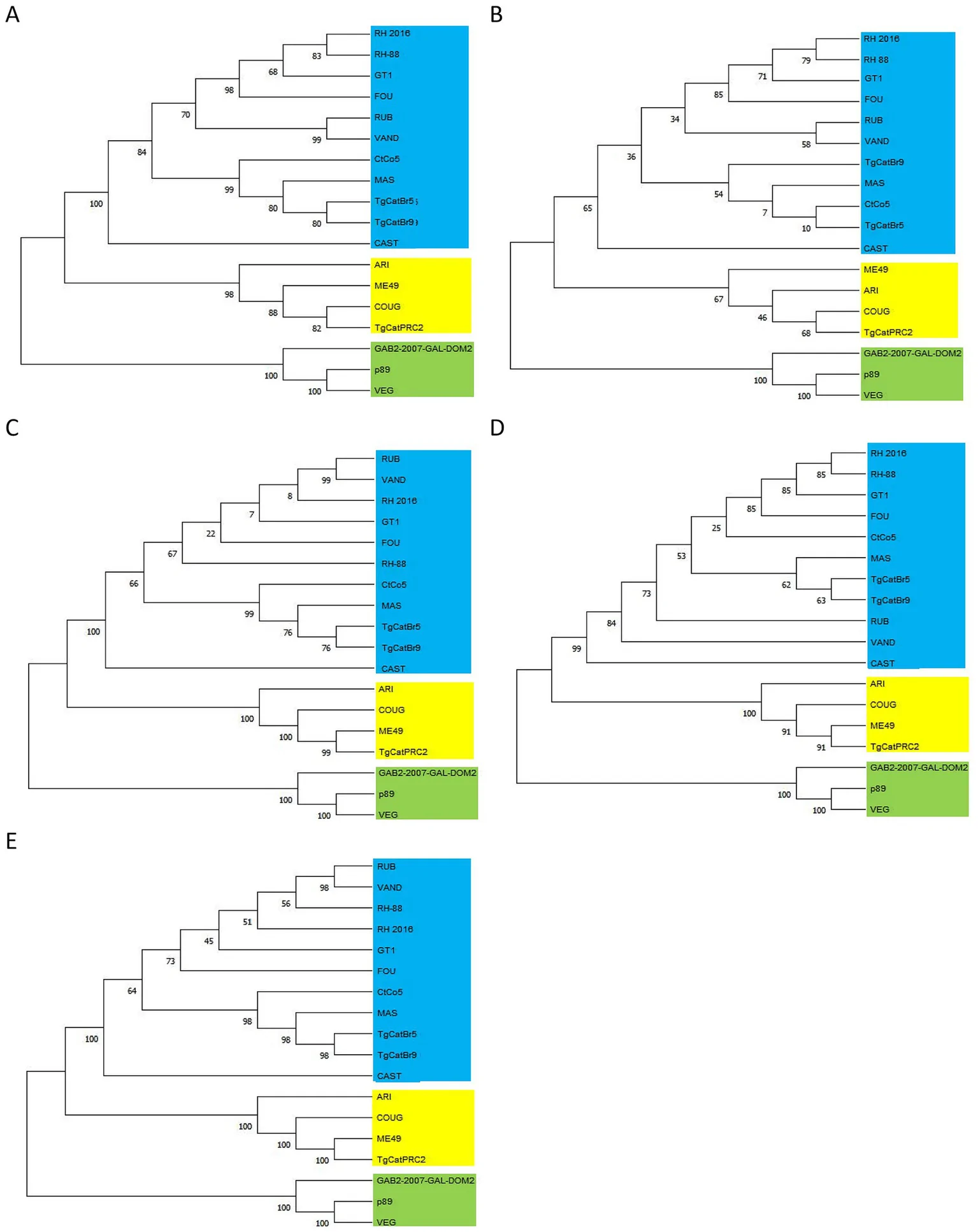
Phylogenetic analysis of *T. gondii str*ains based on different ROP18-associated sequence regions. Phylogenetic trees were constructed based on different ROP18-associated nucleotide and protein sequences extracted from representative *T. gondii str*ains with defined genetic backgrounds. **(A)** Phylogenetic tree constructed using extracted partial genomic sequences surrounding the ROP18 locus. **(B)** Phylogenetic tree based on the ROP18 5′ flanking region. **(C)** Phylogenetic tree based on the ROP18 coding sequence (CDS) region. **(D)** Phylogenetic tree based on the ROP18 3′ flanking region. **(E)** Phylogenetic tree based on the ROP18 protein sequences. Strains are color-coded with different groups indicated in blue, yellow, and green, respectively. Phylogenetic trees were generated using the Neighbor-Joining method with 1,000 bootstrap replicates, and bootstrap values are shown at the corresponding nodes. The different ROP18-associated regions exhibited variable phylogenetic patterns among the analyzed *T. gondii* strains.

To characterize the genetic diversity of the ROP18 locus among representative *T. gondii* strains, genomic sequences covering the 5′ flanking region, ROP18 CDS, and 3′ flanking region, as well as the corresponding protein sequences, were analyzed by phylogenetic approaches. The analyzed sequences and accession information are summarized in Table 2, with the complete sequence dataset provided in Supplementary Sheet 1. Phylogenetic trees were generated using partial genomic sequences (Figure 2A), 5′ flanking regions (Figure 2B), ROP18 CDS regions (Figure 2C), 3′ flanking regions (Figure 2D), and ROP18 protein sequences (Figure 2E).

The analyzed *T. gondii* strains were consistently clustered into three major groups, represented by different colors (blue, yellow, and green). The blue cluster mainly contained RH 2016, RH-88, GT1, FOU, RUB, VAND, CtCo5, MAS, TgCatBr5, TgCatBr9, and CAST. The yellow cluster contained ME49, ARI, COUG, and TgCatPRC2. The green cluster consisted of Type III-related strains, including VEG, p89, and GAB2-2007-GAL-DOM2. Consistent with previous reports, ROP18 alleles in *T. gondii* strains can be broadly categorized into three major allele types based on their sequence characteristics (Khan et al., 2009). According to the ROP18 allele classification reported by Khan et al. (2009), strains carrying ROP18 allele type I, including CAST, CtCo5, FOU, GT1, MAS, RUB, TgCatBr9, and VAND, were grouped within the same blue cluster in our phylogenetic analysis. ME49, previously reported to harbor a ROP18 allele type II, formed a separate yellow cluster. In addition, p89 and VEG, reported to carry ROP18 allele type III, clustered together within the green cluster. These results indicate that the ROP18-based phylogenetic clustering observed in Figure 2 was largely consistent with previously described ROP18 allele classifications among classical *T. gondii* strains.

**Figure 3:**
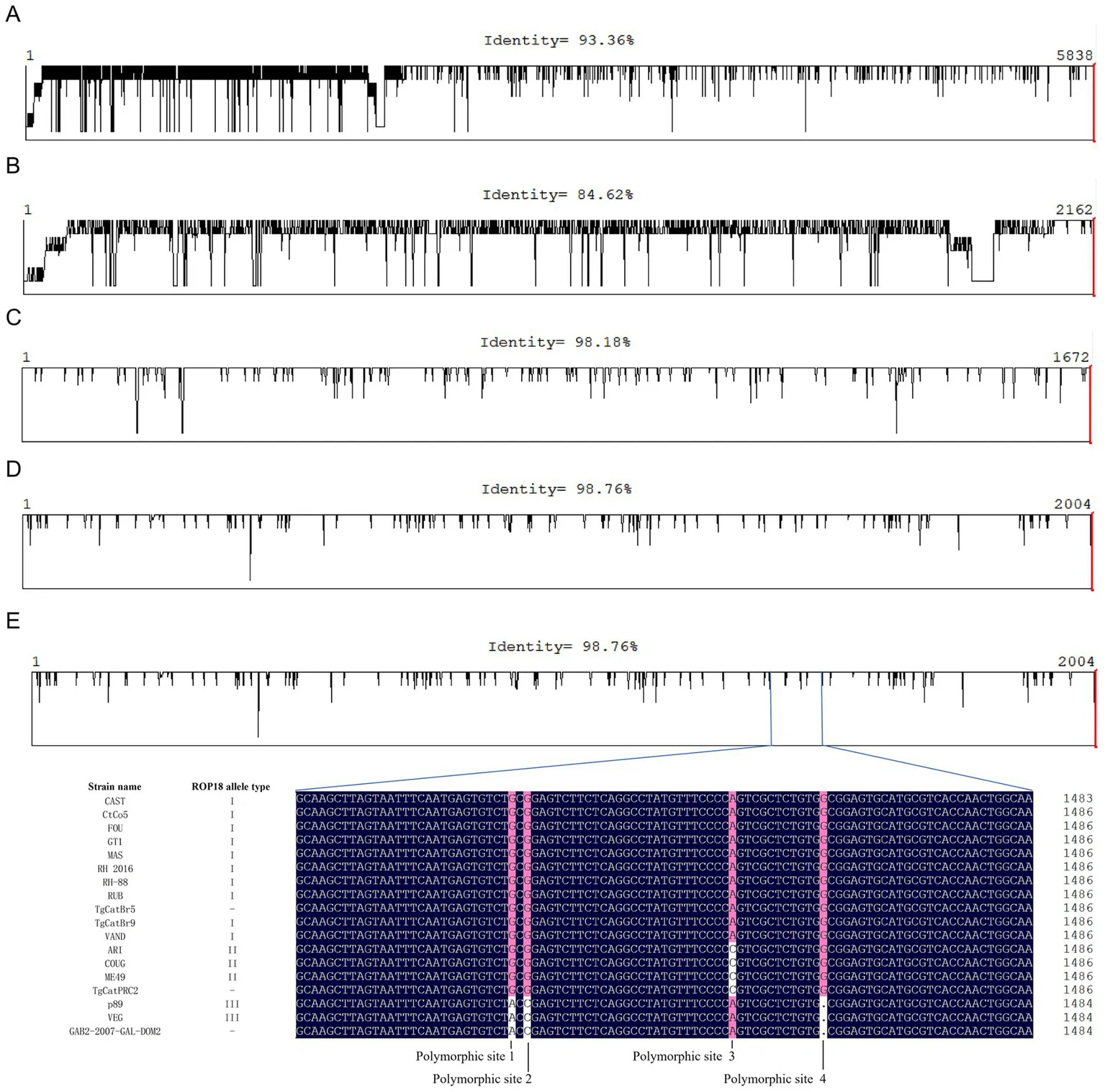
Identification of a short conserved sequence region containing lineage-associated polymorphic sites within the *ROP18* 3′ flanking region. **(A–D)** Sequence identity plots of different *ROP18*-associated regions, including extracted partial genomic sequences **(A)** 5′ flanking regions **(B)** *ROP18* coding sequence (CDS) regions **(C)** 3′ flanking regions **(D)**. **(E)** The genomic location of the selected conserved sequence region within the ROP18 3′ flanking region and highlights the four informative polymorphic sites.

To further characterize sequence variation within the *ROP18* locus, multiple sequence alignment was performed for the extracted genomic regions. The overall sequence identities of the extracted partial genomic sequences, 5′ flanking regions, *ROP18* CDS regions, and 3′ flanking regions were 93.36%, 84.62%, 98.18%, and 98.76%, respectively (Figures 3A–D). Compared with the relatively conserved CDS and 3′ flanking regions, the 5′ flanking region exhibited greater sequence divergence, indicating substantial variation among different *T. gondii* lineages.

Although the ROP18 3′ flanking region was highly conserved among the analyzed strains, a short conserved sequence region of approximately 96–97 bp containing four informative polymorphic sites including nucleotide substitutions and a single-nucleotide deletion was identified (Figure 3E; Table 5). The combination of these polymorphic sites generated distinct lineage-associated sequence patterns corresponding to the three major phylogenetic clusters. Hereafter, these lineage-associated sequence signatures within the ROP18 3′ flanking region are referred to as “lineage-associated ROP18 patterns.” Notably, the identified short conserved sequence region showed a consistent correspondence with the three major clusters observed in the phylogenetic analysis, supporting its potential as a candidate molecular marker for differentiation of *T. gondii* lineages.

**Table 5:** Identification of lineage-associated ROP18 patterns based on polymorphic signatures within the ROP18 3′ flanking region from ToxoDB.

Lineage-associated ROP18 pattern		Genomic location of the corresponding ROP18 3′ flanking sequence	
Type	sequence (5′-3′)	Strain name	Accession No. and genomic region (length)
I	GCAAGCTTAGTAATTTCAATGAGTGTCTGCGGAGTCTTCTCAGGCCTATGTTTCCCCAGTCGCTCTGTGGCGGAGTGCATGCGTCACCAACTGGCAA	CAST	ML130720:3099029–3099125 (97 bp)
		CtCo5	AKIR01000101:c42673-42577 (97 bp)
		FOU	KN000265:c363268-363172 (97 bp)
		GT1	TGGT1_chrVIIa:c1429932-1429836 (97 bp)
		MAS	KN044642:c366958-366862 (97 bp)
		RH 2016	LLKL01000076:204029-204125 (97 bp)
		RH-88	CM023088:c1599036-1598940 (97 bp)
		RUB	KN040001:196331-196427 (97 bp)
		TgCATBr5	AFPV01003518:22777–22873 (97 bp)
		TgCATBr9	KZ791615:c1020891-1020795(97 bp)
		VAND	KN042496:c363875-363779 (97 bp)
II	GCAAGCTTAGTAATTTCAATGAGTGTCTGCGGAGTCTTCTCAGGCCTATGTTTCCCCCGTCGCTCTGTGGCGGAGTGCATGCGTCACCAACTGGCAA	ARI	KQ973610:c1577387-1577291 (97 bp)
		COUG	KZ624886:294825-294921 (97 bp)
		ME49	NC_031474:c1517599-1517503 (97 bp)
		TgCatPRC2	KQ983279:1090358–1090454 (97 bp)
III	GCAAGCTTAGTAATTTCAATGAGTGTCTACCGAGTCTTCTCAGGCCTATGTTTCCCCAGTCGCTCTGTGCGGAGTGCATGCGTCACCAACTGGCAA	p89	KL998096:c1033741-1033646 (96 bp)
		VEG	TGVEG_chrVIIa:c1587966-1587871 (96 bp)
		GAB2-2007-GAL-DOM2	KN003127:c373181-373086 (96 bp)

### 3.2 Validation of lineage-associated ROP18 patterns using newly generated genomes and public sequencing datasets

To further validate the lineage specificity of the identified ROP18 patterns, three representative *T. gondii* genome sequences generated in our previous study and deposited in GenBank, including RH (GenBank accession no. OQ376745), Pru (OQ376746), and CTG (OQ376747), were analyzed. The corresponding ROP18 3′ flanking regions were extracted and compared with the three predefined lineage-associated ROP18 patterns. As shown in Table 6, each of the three representative strains contained one specific lineage-associated ROP18 pattern, with RH carrying the Type I pattern, Pru carrying the Type II pattern, and CTG carrying the Type III pattern.

**Table 6:** Identification of lineage-associated ROP18 patterns in representative *T. gondii str*ains deposited in GenBank.

Strain	GenBank accession No. and genomic coordinates (length)	Lineage-associated ROP18 pattern	Pattern region (length)
RH	OQ376745:1-3,559 (3,559 bp)	I	OQ376745:3112–3,208 (97 bp)
Pru	OQ376746:1-3,559 (3,559 bp)	II	OQ376746:3112–3,208 (97 bp)
CTG	OQ376747:1-3,563 (3,563 bp)	III	OQ376747:3117–3,212 (96 bp)

Furthermore, to investigate the distribution of these lineage-associated ROP18 patterns among a broader collection of *T. gondii* strains, publicly available sequencing datasets were screened using BLAST analysis against the identified ROP18 3′ flanking patterns. Among the analyzed genome datasets retrieved from the SRA databases, each strain contained only one of the three predefined lineage-associated ROP18 patterns and none of the strains simultaneously harbored two or three different patterns. The identified patterns were assigned as Type I-, Type II-, or Type III-associated ROP18 patterns according to their sequence characteristics. The results from 48 *T. gondii* strains are summarized in Table 7, and additional 28 strains are presented in Supplementary Table S1.

**Table 7:** Identification and distribution of lineage-associated ROP18 patterns among *T. gondii str*ains based on publicly available SRA datasets.

No.	Strain name	SRA accession number(s)	Assigned lineage-associated ROP18 pattern
1	ARI	SRX099777 SRX099800	II
2	B41	SRX099774	II
3	B73	SRX159844	III
4	BRC TgH 18009	SRX171132	I
5	BRC TgH 18021	SRX160041	I
6	BRC TgH 26044	SRX160050	III
7	CAST	SRX099788	I
8	COUG	SRX099786 SRX099802 SRX099803	II
9	CtCo5	SRX156155 SRX156164 SRX156192	I
10	FOU	SRX038725 SRX046277 SRX046278	I
11	G662M	SRX160052	III
12	GAB1-2007-GAL-DOM10	SRX159841	III
13	GAB2-2007-GAL-DOM2	SRX155534 SRX155963 SRX156037	III
14	GAB3-2007-GAL-DOM9	SRX160125	III
15	GAB5-2007-GAL-DOM1	SRX160069	I
16	GAB5-2007-GAL-DOM6	SRX159842	III
17	GT1	DRX496953 SRX156314	I
18	GUY-2004-JAG1	SRX099776	II
19	GUY-DOS	SRR446917	I
20	GUY-KOE	SRR446918	I
21	GUY-MAT	SRX099783	I
22	M7741	SRX159890	III
23	MAS	SRX038728 SRX101286	I
24	ME49	DRX496951 SRX055410 SRX17488296 SRX3752882	II
25	p89	SRX038693 SRX038727	III
26	Pru	SRX099792	II
27	RAY	SRX099793	II
28	RH	SRX14879082	I
29	RH-88	SRX160127 SRX8008876	I
30	RH-JSR	SRX159851	I
31	ROD	SRX160129	III
32	RUB	SRX055412 SRX055414 SRX055419 SRX099773	I
33	SOU	SRX160119	II
34	TgCatBr1	SRX099790	I
35	TgCatBr10	SRX099791	I
36	TgCatBr18	SRX099794	I
37	TgCatBr34	SRX160051	I
38	TgCatBr44	SRX160141	I
39	TgCatBr5	SRX099795 SRX099804 SRX099805	I
40	TgCatBr72	SRX160049	I
41	TgCatBr**9**	SRX099781 SRX099806 SRX099807	I
42	TgCatPRC2	SRX155517 SRX156168	II
43	TgCkGy2	SRX099785	III
44	TgDogCo17	SRX099787	I
45	TgRsCr1	SRX160143	III
46	TgShUS28	SRX160130	I
47	VAND	SRX038726 SRX055416 SRX055418	I
48	VEG	SRX156300	III

Collectively, these findings demonstrate that the ROP18 3′ flanking region contains stable lineage-associated sequence signatures that are consistently distributed among diverse *T. gondii* strains, supporting its potential application as a molecular marker for lineage differentiation.

### 3.3 Phylogenetic analysis of lineage-associated ROP18 patterns using 50 *T. Gondii* ROP18-associated genomic sequences

To further evaluate the distribution and conservation of the identified lineage-associated ROP18 patterns, a comprehensive dataset of *T. gondii* genomes was established. First, 48 ROP18-associated genomic sequence regions were recovered from publicly available SRA sequencing datasets listed in Table 7. The ROP18-containing genomic regions were recovered using the representative ROP18-associated sequences derived from the RH, Pru, and CTG (Table 6) as references for read retrieval and targeted sequence recovery.

In addition, 18 publicly available *T. gondii* ROP18-associated genomic sequences obtained from the ToxoDB database (Table 2) were included. After combining these datasets and removing duplicate strains, a final non-redundant dataset comprising 50 representative *T. gondii* ROP18-associated genomic sequences was obtained for subsequent ROP18 pattern analysis (Table 8). The recovered ROP18-associated genomic sequences derived from SRA datasets are provided in Supplementary Sheet 2.

**Table 8:** ROP18-associated genomic sequences from 50 representative *T. gondii str*ains used for ROP18 locus analysis and phylogenetic analysis.

No.	Strain	length (bp)	Deduced CDS region (length)	3′ flank region (length)	Matched sequences from ToxoDB or GenBank
No. 1–48					
1	ARI	3,559	58–1722 (1,665 bp)	1723–3,559 (1837 bp)	KQ973610:c1575958-1572400 (3,559 bp)
2	B41	3,559	58–1722 (1,665 bp)	1723–3,559 (1837 bp)	–
3	B73	3,563	58–1728 (1,671 bp)	1729–3,563 (1835 bp)	–
4	BRC TgH 18,009	3,559	58–1722 (1,665 bp)	1723–3,559 (1837 bp)	–
5	BRC TgH 18,021	3,559	58–1722 (1,665 bp)	1723–3,559 (1837 bp)	–
6	BRC TgH 26,044	3,563	58–1728 (1,671 bp)	1729–3,563 (1835 bp)	–
7	CAST	3,556	58–1722 (1,665 bp)	1723–3,556 (1834 bp)	ML130720:3095921–3,099,476 (3,556 bp)
8	COUG	3,559	58–1722 (1,665 bp)	1723–3,559 (1837 bp)	KZ624886:291714–295,272 (3,559 bp)
9	CtCo5	3,559	58–1722 (1,665 bp)	1723–3,559 (1837 bp)	AKIR01000101:c41244-37686 (3,559 bp)
10	FOU	3,559	58–1722 (1,665 bp)	1723–3,559 (1837 bp)	KN000265:c361839-358281 (3,559 bp)
11	G662M	3,563	58–1728 (1,671 bp)	1729–3,563 (1835 bp)	–
12	GAB1-2007-GAL-DOM10	3,563	58–1728 (1,671 bp)	1729–3,563 (1835 bp)	–
13	GAB2-2007-GAL-DOM2	3,563	58–1728 (1,671 bp)	1729–3,563 (1835 bp)	KN003127:c371754-368192 (3,563 bp)
14	GAB3-2007-GAL-DOM9	3,563	58–1728 (1,671 bp)	1729–3,563 (1835 bp)	–
15	GAB5-2007-GAL-DOM1	3,559	58–1722 (1,665 bp)	1723–3,559 (1837 bp)	–
16	GAB5-2007-GAL-DOM6	3,563	58–1728 (1,671 bp)	1729–3,563 (1835 bp)	–
17	GT1	3,559	58–1722 (1,665 bp)	1723–3,559 (1837 bp)	TGGT1_chrVIIa:c1428503-1424945 (3,559 bp)
18	GUY-2004-JAG1	3,559	58–1722 (1,665 bp)	1723–3,559 (1837 bp)	–
19	GUY-DOS	3,559	58–1722 (1,665 bp)	1723–3,559 (1837 bp)	–
20	GUY-KOE	3,559	58–1722 (1,665 bp)	1723–3,559 (1837 bp)	–
21	GUY-MAT	3,559	58–1722 (1,665 bp)	1723–3,559 (1837 bp)	–
22	M7741	3,563	58–1728 (1,671 bp)	1729–3,563 (1835 bp)	–
23	MAS	3,559	58–1722 (1,665 bp)	1723–3,559 (1837 bp)	KN044642:c365529-361971 (3,559 bp)
24	ME49	3,559	58–1722 (1,665 bp)	1723–3,559 (1837 bp)	TGME49_chrVIIa:c1516170-1512612 (3,559 bp)
25	p89	3,563	58–1728 (1,671 bp)	1729–3,562 (1834 bp)	KL998096:c1032314-1028752 (3,563 bp)
26	Pru	3,559	58–1722 (1,665 bp)	1723–3,559 (1837 bp)	GenBank OQ376746:1–3,559 (3,559 bp)
27	RAY	3,559	58–1722 (1,665 bp)	1723–3,559 (1837 bp)	–
28	RH	3,559	58–1722 (1,665 bp)	1723–3,559 (1837 bp)	GenBank OQ376745:1–3,559 (3,559 bp)
29	RH-88	3,559	58–1722 (1,665 bp)	1723–3,559 (1837 bp)	CM023088:c1597607-1594049 (3,559 bp)
30	RH-JSR	3,559	58–1722 (1,665 bp)	1723–3,559 (1837 bp)	–
31	ROD	3,563	58–1728 (1,671 bp)	1729–3,563 (1835 bp)	–
32	RUB	3,559	58–1722 (1,665 bp)	1723–3,559 (1837 bp)	KN040001:193220–196,778 (3,559 bp)
33	SOU	3,559	58–1722 (1,665 bp)	1723–3,559 (1837 bp)	–
34	TgCatBr1	3,559	58–1722 (1,665 bp)	1723–3,559 (1837 bp)	–
35	TgCatBr10	3,559	58–1722 (1,665 bp)	1723–3,559 (1837 bp)	–
36	TgCatBr18	3,559	58–1722 (1,665 bp)	1723–3,559 (1837 bp)	–
37	TgCatBr34	3,559	58–1722 (1,665 bp)	1723–3,559 (1837 bp)	–
38	TgCatBr44	3,559	58–1722 (1,665 bp)	1723–3,559 (1837 bp)	–
39	TgCATBr5	3,559	58–1722 (1,665 bp)	1723–3,559 (1837 bp)	AFPV01003518:19666–23,224 (3,559 bp)
40	TgCatBr72	3,559	58–1722 (1,665 bp)	1723–3,559 (1837 bp)	–
41	TgCATBr9	3,559	58–1722 (1,665 bp)	1723–3,559 (1837 bp)	KZ791615:c1019462-1015904 (3,559 bp)
42	TgCatPRC2	3,559	58–1722 (1,665 bp)	1723–3,559 (1837 bp)	KQ983279:1087247–1,090,805 (3,559 bp)
43	TgCkGy2	3,563	58–1728 (1,671 bp)	1729–3,563 (1835 bp)	–
44	TgDogCo17	3,559	58–1722 (1,665 bp)	1723–3,559 (1837 bp)	–
45	TgRsCr1	3,545	58–1728 (1,671 bp)	1729–3,545 (1817 bp)	–
46	TgShUS28	3,559	58–1722 (1,665 bp)	1723–3,559 (1837 bp)	–
47	VAND	3,559	58–1722 (1,665 bp)	1723–3,559 (1837 bp)	KN042496:c362446-358888 (3,559 bp)
48	VEG	3,563	58–1728 (1,671 bp)	1729–3,563 (1835 bp)	TGVEG_chrVIIa:c1586539-1582977 (3,563 bp)
No. 49					
49	CTG	3,563	58–1728 (1,671 bp)	1729–3,563 (1835 bp)	GenBank OQ376747:1–3,563 (3,563 bp)
No. 50					
50	RH 2016	3,559	200,975–202,639 (1,665 bp)	202,640–204,476 (1837 bp)	LLKL01000076:200918–204,476 (3,559 bp)

No. 1–48 represent genome assemblies recovered from publicly available SRA datasets, including some strains with previously reported genome sequences in GenBank or ToxoDB. No. 49 is the CTG genome sequence deposited by this study in GenBank (accession no. OQ376747), and No. 50 is the RH 2016 reference genome obtained from ToxoDB.

**Figure 4:**
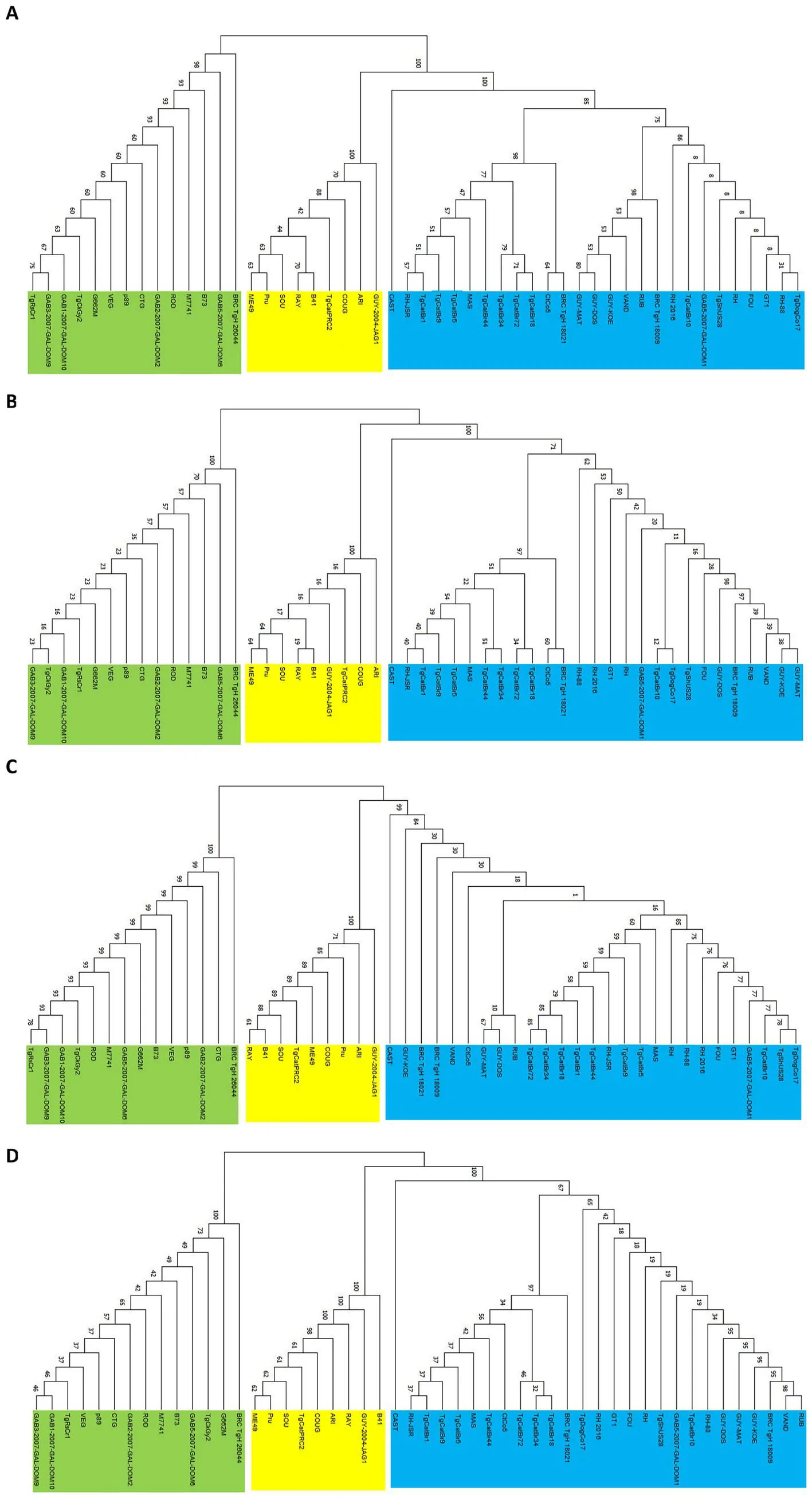
Phylogenetic analysis of 50 representative *T. gondii* strains based on different ROP18-associated sequence regions. Phylogenetic trees were constructed using four different ROP18-associated sequence datasets: **(A)** extracted partial genomic sequences encompassing the ROP18 locus; **(B)** ROP18 coding sequences (CDS); **(C)** ROP18 3′ flanking regions; and **(D)** deduced ROP18 protein sequences. Phylogenetic trees were generated using the Neighbor-Joining method with 1,000 bootstrap replicates, and bootstrap support values are indicated at the corresponding nodes. The three major clusters identified in the phylogenetic analyses are highlighted in different colors according to their lineage-associated ROP18 patterns: blue, Type I-associated pattern; yellow, Type II-associated pattern; and green, Type III-associated pattern.

Phylogenetic analyses were subsequently performed using ROP18-associated genomic sequences from 50 *T. gondii* strains to further evaluate the distribution and consistency of the identified lineage-associated ROP18 patterns. Multiple sequence datasets, including extracted partial genomic sequences encompassing the ROP18 locus, ROP18 coding sequences (CDS), ROP18 3′ flanking regions, and the corresponding deduced ROP18 protein sequences, were analyzed. As shown in Figures 4A–D, phylogenetic trees constructed from different ROP18-associated sequence regions consistently separated the 50 *T. gondii* strains into three major clusters, which were represented by different colors. Importantly, strains grouped within the same color-coded cluster shared identical lineage-associated ROP18 patterns, which were consistent with the pattern assignments predicted from the sequence analysis shown in the corresponding tables. Furthermore, the color-coded clustering patterns observed in Figure 4 were highly concordant with those identified in the representative strain analysis shown in Figure 2, indicating that the expanded genome dataset reproduced the clustering characteristics observed in the initial phylogenetic analysis.

The clustering patterns derived from the extracted genomic regions, ROP18 CDS regions, 3′ flanking regions, and deduced protein sequences showed overall concordance. These findings indicate that the polymorphic signatures identified within the ROP18 3′ flanking region were consistent with the broader evolutionary relationships among the analyzed *T. gondii* strains. Therefore, the ROP18 3′ flanking region represents an informative genomic region for differentiation of *T. gondii* strains based on lineage-associated ROP18 patterns.

### 3.4 Development and validation of an HRM-PCR assay for differentiation of lineage-associated ROP18 patterns

The three identified lineage-associated ROP18 patterns were further evaluated for their suitability for HRM-PCR-based genotyping. The lengths of the Type I-, Type II-, and Type III-associated ROP18 patterns were 97 bp, 97 bp, and 96 bp, respectively, with GC contents of 52.58%, 53.61%, and 51.04%. These sequence characteristics indicated that the three patterns possessed appropriate nucleotide compositions for HRM-based differentiation (Martino et al., 2010).

**Figure 5:**
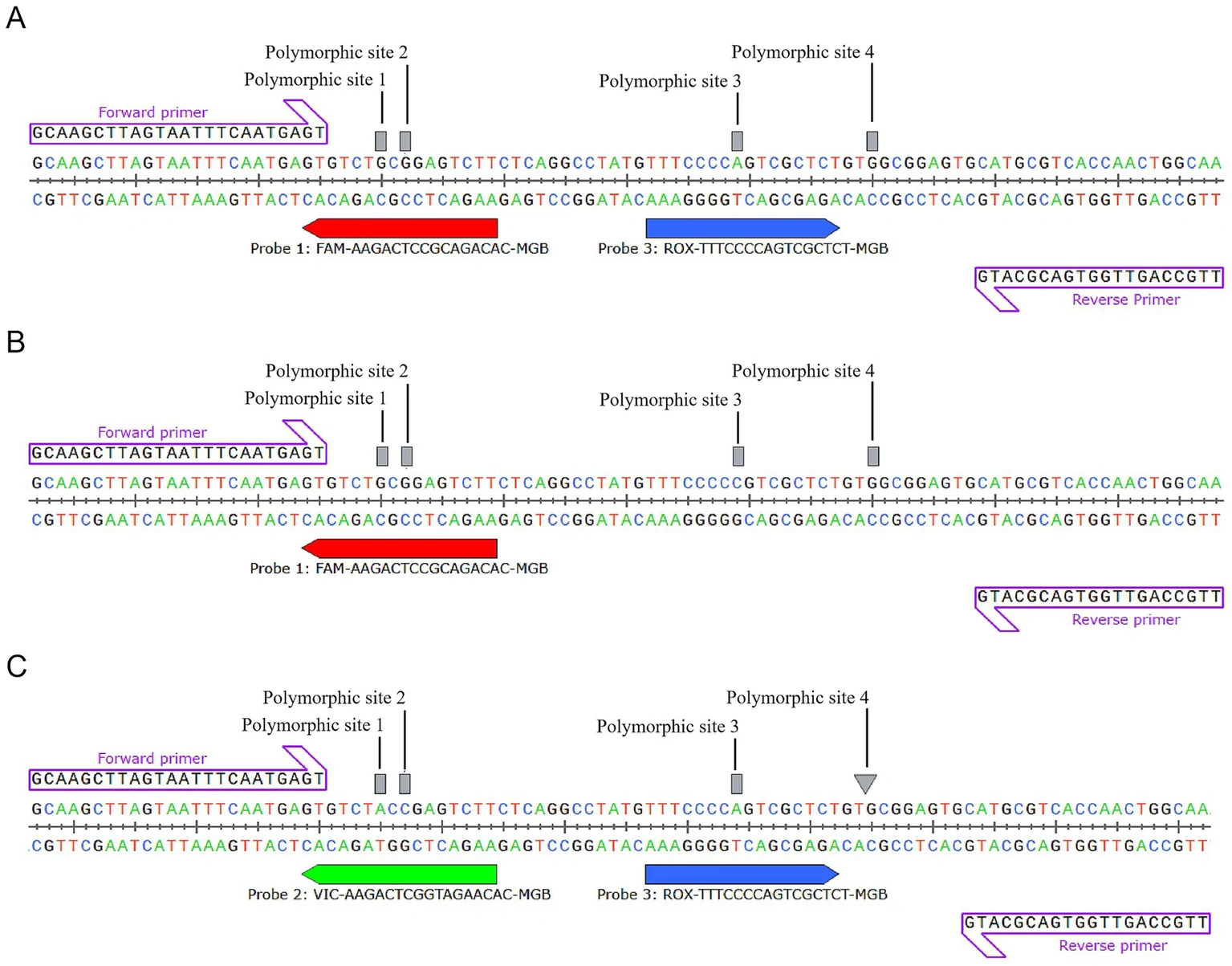
Design of HRM-PCR primers and TaqMan MGB probes targeting lineage-associated polymorphic sites within the ROP18 3′ flanking region. The relative positions of the forward primer, reverse primer, four informative polymorphic sites, and TaqMan MGB probes are indicated within the three identified ROP18 3′ flanking region patterns: **(A)** Type I-associated ROP18 pattern; **(B)** Type II-associated ROP18 pattern; and **(C)** Type III-associated ROP18 pattern. Three fluorescent MGB probes labeled with FAM, VIC, and ROX were designed to detect the corresponding pattern-associated sequence signatures within the ROP18 3′ flanking region.

To establish HRM-PCR reference standards, plasmids containing the three lineage-associated ROP18 patterns were commercially synthesized (Figure 1). These plasmids were used as known genotype controls representing Type I, Type II, and Type III lineage-associated ROP18 patterns. Specific primers were designed according to the conserved regions flanking the lineage-associated SNP loci, and the positions of the primers and probes relative to the target sequence are shown in Figure 5.

The specificity of the HRM-PCR assay was initially evaluated using plasmid controls and representative *T. gondii* genomic DNA. All three lineage-associated ROP18 patterns were specifically amplified, whereas no detectable amplification was observed in the negative control, demonstrating the specificity of the established assay (Supplementary Figure S1).

**Figure 6:**
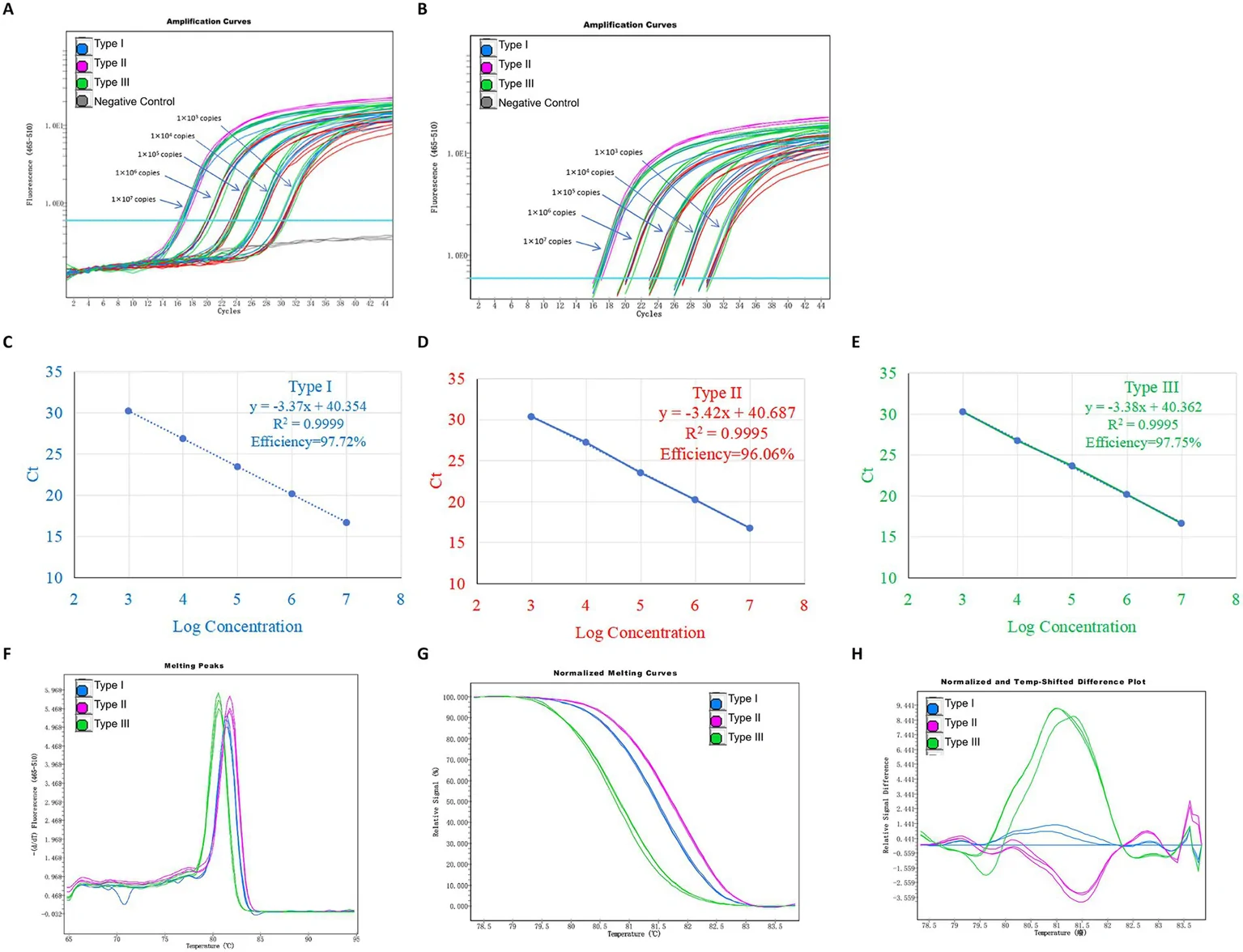
Validation of the HRM-PCR assay for differentiation of lineage-associated ROP18 patterns using synthetic plasmid standards. Synthetic plasmids containing representative Type I-, Type II-, and Type III-associated ROP18 pattern sequences were serially diluted and used as defined DNA templates for HRM-PCR assay validation. Double-distilled water (ddH_2_O) was included as the no-template negative control. **(A,B)** Amplification curves generated by HRM-PCR using serially diluted plasmid standards representing the three ROP18 patterns. The plasmid concentrations ranged from 1 × 10^3^ to 1 × 10^7^ copies/reaction. No amplification signal was detected in the negative controls containing ddH_2_O. **(C–E)** Standard curves generated from serial dilutions of Type I-, Type II-, and Type III-associated ROP18 pattern plasmid standards, respectively. The corresponding linear regression equations, correlation coefficients (*R*^2^), and amplification efficiencies are shown. **(F)** Melting peak analysis of HRM-PCR products generated from the three plasmid standards, showing distinct melting profiles among the different ROP18 patterns. **(G)** Normalized melting curves demonstrating differential melting behaviors among Type I-, Type II-, and Type III-associated ROP18 patterns. **(H)** Normalized and temperature-shifted difference plots showing clear separation of the three ROP18 pattern groups.

Analytical performance of the HRM-PCR assay was further evaluated using serially diluted plasmid standards. The three lineage-associated ROP18 patterns were successfully amplified across the tested concentration range (Figures 6A,B). Standard curves demonstrated excellent linearity among different plasmid concentrations, with high correlation coefficients and amplification efficiencies. The amplification efficiencies were 97.72, 96.06, and 97.75% for the Type I-, Type II-, and Type III-associated ROP18 patterns, respectively, with excellent linearity (*R*^2^ = 0.9999, 0.9995, and 0.9995, respectively), indicating consistent amplification performance across the tested concentration range. (Figures 6C–E). Furthermore, melting curve analysis and normalized high-resolution melting profiles showed clear differentiation among Type I-, Type II-, and Type III-associated ROP18 patterns (Figures 6F–H).

**Figure 7:**
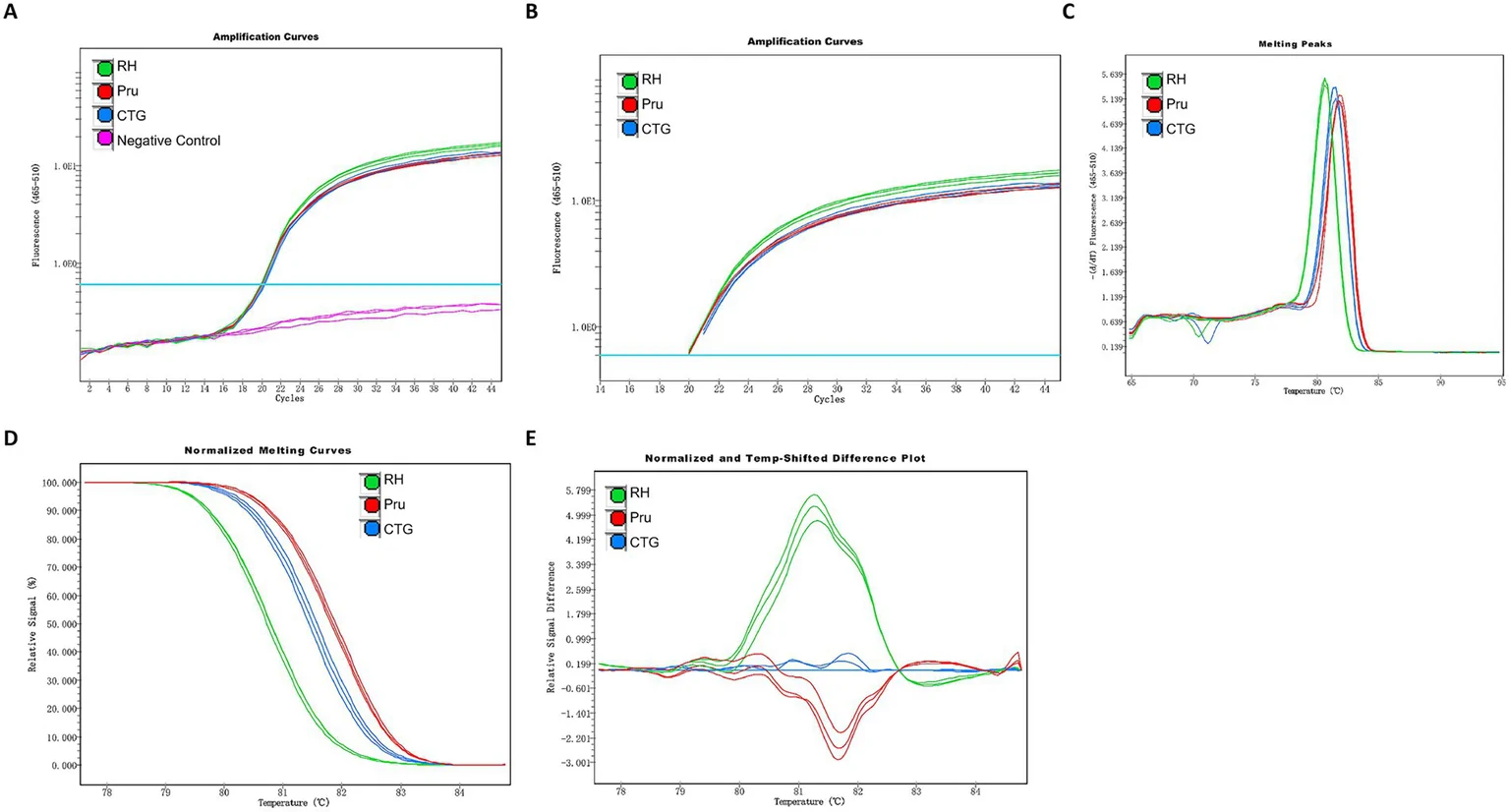
Validation of the HRM-PCR assay for differentiation of lineage-associated ROP18 patterns using *T. gondii gen*omic DNA. Genomic DNA extracted from representative *T. gondii str*ains, including RH (Type I-associated ROP18 pattern), Pru (Type II-associated ROP18 pattern), and CTG (Type III-associated ROP18 pattern), was used to further validate the HRM-PCR assay. Double-distilled water (ddH_2_O) was included as the no-template negative control. **(A,B)** Amplification curves generated by HRM-PCR using genomic DNA templates from RH, Pru, and CTG strains. All three representative strains produced specific amplification signals, whereas no amplification signal was detected in the negative control. **(C)** Melting peak analysis of HRM-PCR products derived from RH, Pru, and CTG genomic DNA, showing distinct melting peak profiles among the three ROP18 pattern groups. **(D)** Normalized melting curves demonstrating differential melting behaviors of the Type I-, Type II-, and Type III-associated ROP18 patterns. **(E)** Normalized and temperature-shifted difference plots showing clear separation of RH-, Pru-, and CTG-derived ROP18 pattern groups.

The HRM-PCR assay was further validated using genomic DNA from representative *T. gondii* strains, including RH, Pru, and CTG. All genomic DNA samples showed specific amplification signals, and no amplification was detected in negative controls (Figures 7A,B). HRM analysis generated distinct melting peak profiles, normalized melting curves, and difference plots among RH-, Pru-, and CTG-derived templates, demonstrating reliable differentiation of the three ROP18-associated patterns (Figures 7C–E).

### 3.5 Intra-assay repeatability and inter-assay reproducibility of the HRM-PCR assay

The repeatability and reproducibility of the established HRM-PCR assay were evaluated using recombinant plasmid standards representing the three ROP18-associated patterns and genomic DNA samples from representative *T. gondii* strains (RH, Pru, and CTG). As shown in Table 9, the assay demonstrated stable amplification performance across a wide concentration range of plasmid templates (1 × 10^3^ to 1 × 10^7^ copies/reaction). For the three plasmid standards, intra-assay and inter-assay analyses showed low variation in Ct values among technical replicates and independent experiments. The coefficients of variation (CV%) for intra-assay analysis ranged from 0.69% to 2.43% for Type I-associated pattern, 0.69% to 2.09% for Type II-associated pattern, and 0.97% to 2.15% for Type III-associated pattern, respectively. The corresponding inter-assay CV values ranged from 1.03% to 2.50%, 1.19% to 1.79%, and 0.71% to 2.10%, respectively. Similar reproducibility was observed when genomic DNA templates were analyzed, with low intra-assay and inter-assay variation among RH, Pru, and CTG samples.

**Table 9:** Intra-assay repeatability and inter-assay reproducibility of Ct values for the HRM-PCR assay using plasmid standards and genomic DNA templates.

Sample		Intra-assay repeatability			Inter-assay reproducibility		
		Mean Ct	SD	CV%	Mean Ct	SD	CV%
Plasmid	Copy number						
Type I	1 × 10^3^	30.21	0.38	1.26	30.29	0.31	1.03
	1 × 10^4^	26.85	0.21	0.78	27.10	0.34	1.26
	1 × 10^5^	23.44	0.57	2.43	23.64	0.46	1.94
	1 × 10^6^	20.16	0.26	1.29	20.21	0.24	1.17
	1 × 10^7^	16.67	0.26	1.55	16.57	0.41	2.50
Type II	1 × 10^3^	30.33	0.21	0.69	30.48	0.36	1.19
	1 × 10^4^	27.20	0.57	2.09	27.12	0.48	1.79
	1 × 10^5^	23.49	0.26	1.11	23.52	0.39	1.66
	1 × 10^6^	20.19	0.26	1.28	20.04	0.25	1.22
	1 × 10^7^	16.73	0.22	1.29	16.64	0.21	1.28
Type III	1 × 10^3^	30.24	0.57	1.88	30.53	0.37	1.21
	1 × 10^4^	26.71	0.26	0.97	26.59	0.29	1.10
	1 × 10^5^	23.64	0.26	1.09	23.33	0.38	1.65
	1 × 10^6^	20.16	0.22	1.07	20.00	0.14	0.71
	1 × 10^7^	16.63	0.36	2.15	16.57	0.35	2.10
Genomic DNA							
RH		19.81	0.17	0.86	19.57	0.21	1.05
Pru		19.67	0.05	0.23	19.65	0.24	1.20
CTG		19.58	0.09	0.44	19.80	0.19	0.97

Synthetic plasmids containing Type I-, Type II-, and Type III-associated ROP18 patterns were used as reference controls. Repeatability (intra-assay precision) was evaluated using three technical replicates within the same run, whereas reproducibility (inter-assay precision) was assessed using three independent experiments performed on separate days. Mean Ct values, standard deviations (SD), and coefficients of variation (CV%) were calculated.

In addition to amplification reproducibility, the stability of HRM melting profiles was further assessed by comparing the melting temperature (Tm) values between intra-assay and inter-assay experiments (Table 10). Plasmid standards representing Type I-, Type II-, and Type III-associated ROP18 patterns showed highly consistent melting temperatures, with mean Tm values of 81.45 °C ± 0.05 °C, 81.81 °C ± 0.04 °C, and 80.65 °C ± 0.05 °C in intra-assay analysis, respectively. Similar Tm values were obtained in inter-assay experiments (81.48 °C ± 0.03 °C, 81.80 °C ± 0.02 °C, and 80.64 °C ± 0.06 °C, respectively). The corresponding genomic DNA samples exhibited comparable melting profiles, with RH, Pru, and CTG showing mean Tm values of 81.45 °C ± 0.03 °C, 81.82 °C ± 0.04 °C, and 80.64 °C ± 0.06 °C, respectively. Similar Tm values were obtained in inter-assay experiments for the corresponding genomic DNA samples (81.44 °C ± 0.05 °C, 81.80 °C ± 0.04 °C, and 80.68 °C ± 0.03 °C, respectively). The CV values of Tm measurements remained extremely low (0.02%–0.07%), indicating excellent stability of the HRM melting profiles. Although the mean Tm difference between Type I- and Type II-associated patterns was relatively small, these patterns showed reproducible differences in normalized melting behavior and difference plot clustering.

**Table 10:** Intra-assay repeatability and inter-assay reproducibility of melting temperatures for HRM-PCR differentiation of ROP18-associated patterns.

Sample	Intra-assay repeatability			Inter-assay reproducibility		
	Mean Tm (°C)	SD	CV%	Mean Tm (°C)	SD	CV%
Plasmid						
Type I	81.45	0.05	0.06	81.48	0.03	0.03
Type II	81.81	0.04	0.05	81.80	0.02	0.02
Type III	80.65	0.05	0.06	80.64	0.06	0.07
Genomic DNA						
RH	81.45	0.03	0.04	81.44	0.05	0.06
Pru	81.82	0.04	0.05	81.81	0.04	0.05
CTG	80.64	0.06	0.07	80.68	0.03	0.04

Tm values are presented as mean ± SD from three technical replicates. RH, Pru, and CTG represent Type I-, Type II-, and Type III-associated ROP18 patterns, respectively.

Collectively, these results demonstrate that the HRM-PCR assay exhibits high amplification precision and highly reproducible melting characteristics, supporting its reliability for differentiation of different ROP18-associated sequence patterns.

### 3.6 Development and validation of a TaqMan probe-based assay for differentiation of ROP18 lineage-associated patterns

Based on the lineage-associated *ROP18* patterns identified by comparative sequence analysis, three TaqMan MGB probes were designed to enable specific recognition of different genotypic patterns (Figure 5; Table 4). The same primer pair used for HRM-PCR was applied for TaqMan assays, generating amplicons of 97 bp, 97 bp, and 96 bp for Type I-, Type II-, and Type III-associated patterns, respectively.

#### 3.6.1 Validation of probe compatibility with PCR amplification

The compatibility of the designed TaqMan MGB probes with PCR amplification was further evaluated. As shown in Supplementary Figure S2, plasmid standards containing the three ROP18-associated patterns and genomic DNA samples from RH, Pru, and CTG strains generated specific amplicons of the expected size (~100 bp) under the corresponding probe conditions. No amplification was observed using THP-1 or RAW264.7 genomic DNA controls. These results demonstrated that the designed probes did not interfere with amplification of the ROP18 target region.

#### 3.6.2 Validation of the TaqMan MGB probe-based assay for type I-associated ROP18 pattern detection

**Figure 8:**
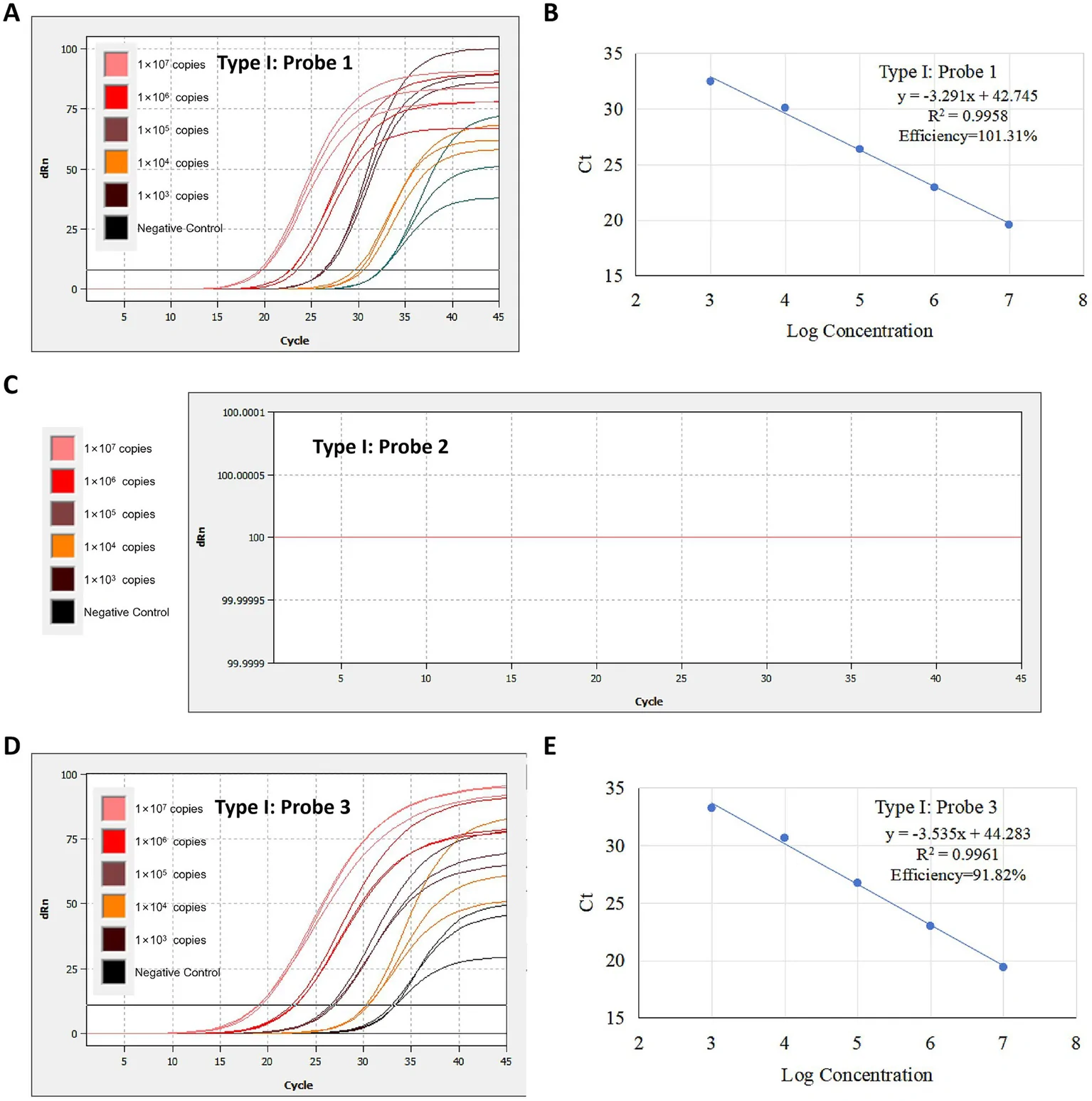
Analytical validation of the TaqMan MGB probe-based assay for detection of the Type I-associated ROP18 pattern. A plasmid standard containing the Type I-associated ROP18 pattern sequence was serially diluted from 1 × 10^7^ to 1 × 10^3^ copies/reaction and analyzed using three designed TaqMan MGB probes. **(A)** Amplification curves generated using Probe 1. **(B)** Standard curve analysis of Probe 1, showing the regression equation, correlation coefficient (*R*^2^), and amplification efficiency. **(C)** Amplification analysis using Probe 2, showing no detectable amplification signal. **(D)** Amplification curves generated using Probe 3. **(E)** Standard curve analysis of Probe 3, showing the regression equation, correlation coefficient (*R*^2^), and amplification efficiency.

To evaluate the analytical performance of the TaqMan MGB probe-based assay for detection of the Type I-associated ROP18 pattern, a recombinant plasmid standard containing the representative Type I-associated ROP18 sequence was serially diluted from 1 × 10^7^ to 1 × 10^3^ copies/reaction and analyzed using the three designed probes. As shown in Figures 8A,C,D, the Type I-associated pattern generated specific amplification signals with Probe 1 and Probe 3, whereas no amplification signal was detected with Probe 2, consistent with the expected probe recognition profile.

Standard curves were generated using the serially diluted plasmid standards for the compatible probes. Probe 1 showed excellent linearity across the tested concentration range, with a regression equation of y = −3.291x + 42.745, an R^2^ value of 0.9958, and an amplification efficiency of 101.31% (Figures 8A,B). Probe 3 also demonstrated a good linear amplification relationship, with a regression equation of y = −3.535x + 44.283, an R^2^ value of 0.9961, and an amplification efficiency of 91.82% (Figures 8D,E). These results indicate that the designed TaqMan MGB probes can specifically recognize the Type I-associated ROP18 pattern with reliable amplification performance.

To further evaluate the reproducibility of probe detection, intra-assay repeatability and inter-assay reproducibility were assessed using serially diluted plasmid standards and RH genomic DNA templates. The mean Ct values, standard deviations (SD), and coefficients of variation (CV%) were calculated (Table 11). For Probe 1, the intra-assay CV values ranged from 0.14% to 1.80%, and the inter-assay CV values ranged from 1.00% to 1.92% across the tested plasmid concentrations (1 × 10^3^–1 × 10^7^ copies/reaction). For Probe 3, the intra-assay and inter-assay CV values ranged from 0.99 to 1.97% and 0.68 to 1.79%, respectively. Similar reproducibility was observed using RH genomic DNA, with low intra-assay and inter-assay variability for both probes. These findings demonstrate the stability and reproducibility of the TaqMan MGB probe-based assay for identification of the Type I-associated ROP18 pattern.

**Table 11:** Intra-assay repeatability and inter-assay reproducibility of the TaqMan MGB probe-based assay for detection of the Type I-associated ROP18 pattern.

Sample	Repeatability			Reproducibility		
	Mean Ct	SD	CV%	Mean Ct	SD	CV%
Probe 1						
Copy number of plasmid						
1 × 10^3^	32.46	0.05	0.14	32.53	0.33	1.00
1 × 10^4^	30.09	0.45	1.51	30.36	0.45	1.49
1 × 10^5^	26.38	0.10	0.37	26.56	0.33	1.24
1 × 10^6^	22.94	0.41	1.80	22.70	0.25	1.10
1 × 10^7^	19.58	0.18	0.93	19.55	0.38	1.92
Genomic DNA of RH	22.49	0.16	0.73	22.36	0.26	1.18
Probe 2						
Copy number of plasmid						
1 × 10^3^	–	–	–	–	–	–
1 × 10^4^	–	–	–	–	–	–
1 × 10^5^	–	–	–	–	–	–
1 × 10^6^	–	–	–	–	–	–
1 × 10^7^	–	–	–	–	–	–
Genomic DNA of RH	–	–	–	–	–	–
Probe 3						
Copy number of plasmid						
1 × 10^3^	32.84	0.33	0.99	32.68	0.33	1.01
1 × 10^4^	30.60	0.42	1.36	30.53	0.21	0.68
1 × 10^5^	26.85	0.42	1.56	26.78	0.35	1.29
1 × 10^6^	23.16	0.34	1.46	23.21	0.24	1.05
1 × 10^7^	19.57	0.38	1.97	19.56	0.35	1.79
Genomic DNA of RH	22.31	0.28	1.24	22.26	0.20	0.90

Repeatability was evaluated using three technical replicates within the same assay run, whereas reproducibility was assessed using three independent experiments performed on different days. Plasmid standards containing lineage-associated *ROP18* patterns were serially diluted from 1 × 10^3^ to 1 × 10^7^ copies/reaction for assay evaluation. Genomic DNA samples from representative *T. gondii* strains (RH, Pru, and CTG) were included for validation. Mean Ct values, standard deviations (SD), and coefficients of variation (CV%) were calculated from replicate measurements. A dash (−) indicates that no amplification signal was detected.

#### 3.6.3 Validation of the TaqMan MGB probe-based assay for type II-associated ROP18 pattern detection

**Figure 9:**
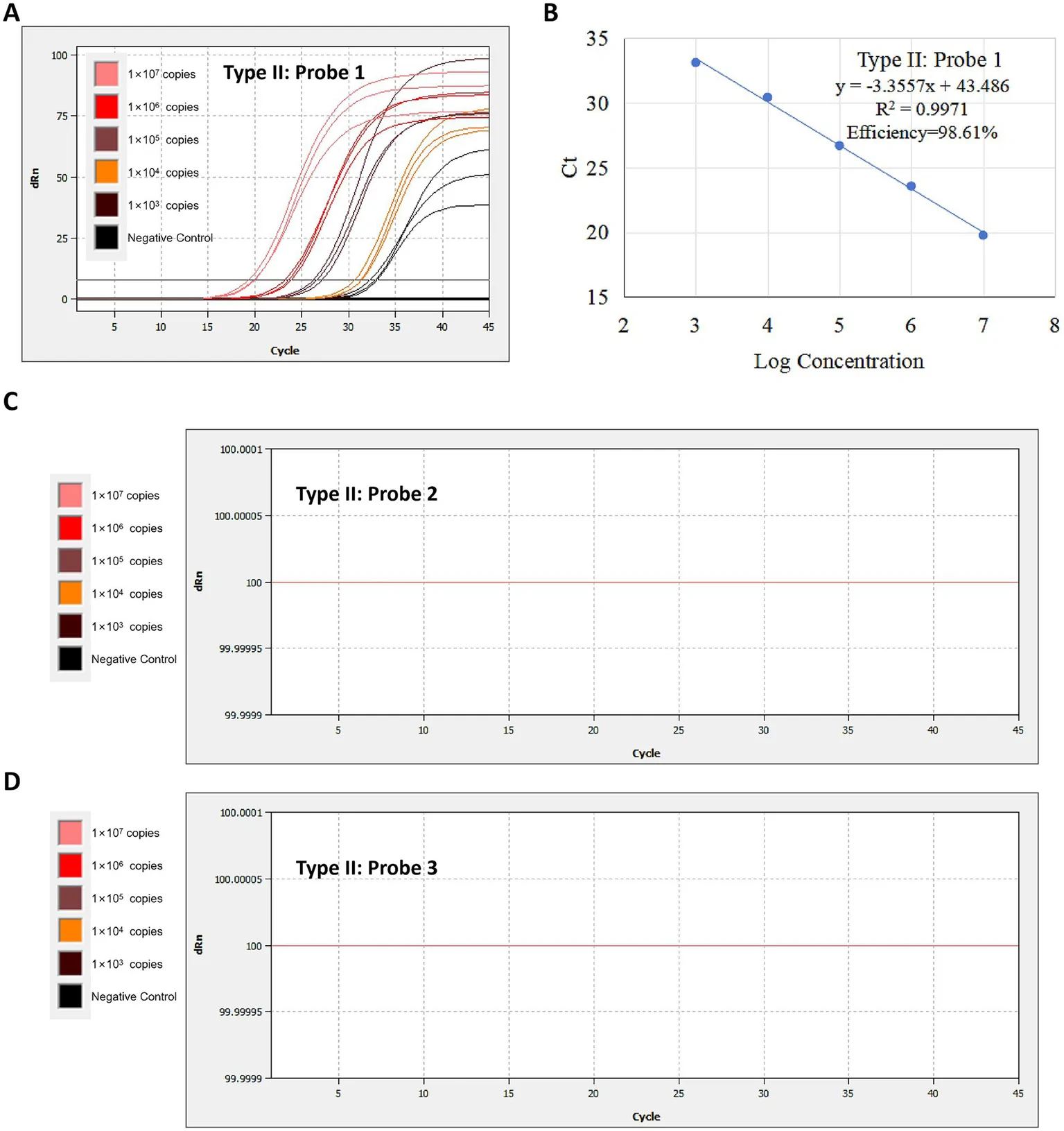
Analytical validation of the TaqMan MGB probe-based assay for detection of the type II-associated ROP18 pattern. A recombinant plasmid containing the type II-associated ROP18 pattern sequence was serially diluted from 1 × 10^7^ to 1 × 10^3^ copies/reaction and analyzed using three designed TaqMan MGB probes. **(A)** Amplification curves generated using probe 1. **(B)** Standard curve analysis of probe 1, showing the regression equation, correlation coefficient (*R*^2^), and amplification efficiency. **(C)** Amplification analysis using probe 2, showing no detectable amplification signal. **(D)** Amplification analysis using probe 3, showing no detectable amplification signal.

To further evaluate the performance of the TaqMan MGB probe-based assay for identification of the Type II-associated ROP18 pattern, a recombinant plasmid containing the representative Type II-associated ROP18 sequence was serially diluted from 1 × 10^7^ to 1 × 10^3^ copies/reaction and analyzed using the three designed TaqMan probes. As shown in Figures 9A,C,D, the Type II-associated pattern produced a specific amplification signal with Probe 1, whereas no amplification signal was detected with Probe 2 or Probe 3, consistent with the expected probe recognition profile.

The standard curve generated using Probe 1 demonstrated a strong linear relationship between Ct values and logarithmic plasmid copy numbers over the tested concentration range. The corresponding regression equation was y = −3.3557x + 43.486, with an R^2^ value of 0.9971 and an amplification efficiency of 98.61% (Figures 9A,B). These results indicate that Probe 1 provides reliable amplification performance for detection of the Type II-associated ROP18 pattern.

The repeatability and reproducibility of Probe 1-based detection were further evaluated using serially diluted plasmid standards and genomic DNA from the representative Type II strain Pru. As summarized in Table 12, the assay showed low intra-assay and inter-assay variability across the tested concentration range. For plasmid standards ranging from 1 × 10^3^ to 1 × 10^7^ copies/reaction, the intra-assay CV values ranged from 0.49% to 1.75%, while the inter-assay CV values ranged from 0.72% to 1.75%. Detection of Pru genomic DNA also showed good precision, with intra-assay and inter-assay CV values of 1.02% and 0.93%, respectively. These results demonstrate the stability, repeatability, and reproducibility of the TaqMan MGB probe-based assay for identification of the Type II-associated ROP18 pattern.

**Table 12:** Intra-assay repeatability and inter-assay reproducibility of the TaqMan MGB probe-based assay for detection of the Type II-associated ROP18 pattern.

Sample	Repeatability			Reproducibility		
	Mean Ct	SD	CV%	Mean Ct	SD	CV%
Probe 1						
Copy number of plasmid						
1 × 10^3^	33.11	0.22	0.67	33.18	0.24	0.72
1 × 10^4^	30.42	0.15	0.49	30.60	0.24	0.80
1 × 10^5^	26.69	0.47	1.75	26.65	0.27	1.02
1 × 10^6^	23.56	0.34	1.45	23.37	0.41	1.75
1 × 10^7^	19.76	0.31	1.58	19.54	0.33	1.70
Genomic DNA of Pru	22.67	0.23	1.02	22.63	0.21	0.93
Probe 2						
Copy number of plasmid						
1 × 10^3^	–	–	–	–	–	–
1 × 10^4^	–	–	–	–	–	–
1 × 10^5^	–	–	–	–	–	–
1 × 10^6^	–	–	–	–	–	–
1 × 10^7^	–	–	–	–	–	–
Genomic DNA of Pru	–	–	–	–	–	–
Probe 3						
Copy number of plasmid						
1 × 10^3^	–	–	–	–	–	–
1 × 10^4^	–	–	–	–	–	–
1 × 10^5^	–	–	–	–	–	–
1 × 10^6^	–	–	–	–	–	–
1 × 10^7^	–	–	–	–	–	–
Genomic DNA of Pru	–	–	–	–	–	–

Repeatability was evaluated using three technical replicates within the same assay run, whereas reproducibility was assessed using three independent experiments performed on different days. A Type II lineage-associated *ROP18* plasmid standard was serially diluted from 1 × 10^3^ to 1 × 10^7^ copies/reaction. Representative genomic DNA from the Pru strain was further analyzed for validation. Mean Ct values, standard deviations (SD), and coefficients of variation (CV%) were calculated from replicate measurements. A dash (−) indicates no detectable amplification signal.

#### 3.6.4 Validation of the TaqMan MGB probe-based assay for type III-associated ROP18 pattern detection

The Type III-associated ROP18 pattern was subsequently evaluated using the established TaqMan MGB probe-based real-time PCR assay. A recombinant plasmid containing the representative Type III-associated ROP18 sequence was serially diluted from 1 × 10^7^ to 1 × 10^3^ copies/reaction and analyzed using the three designed TaqMan probes. According to the predicted probe recognition profile, the Type III-associated pattern was expected to generate positive fluorescence signals with Probe 2 and Probe 3, while no amplification signal should be produced with Probe 1.

**Figure 10:**
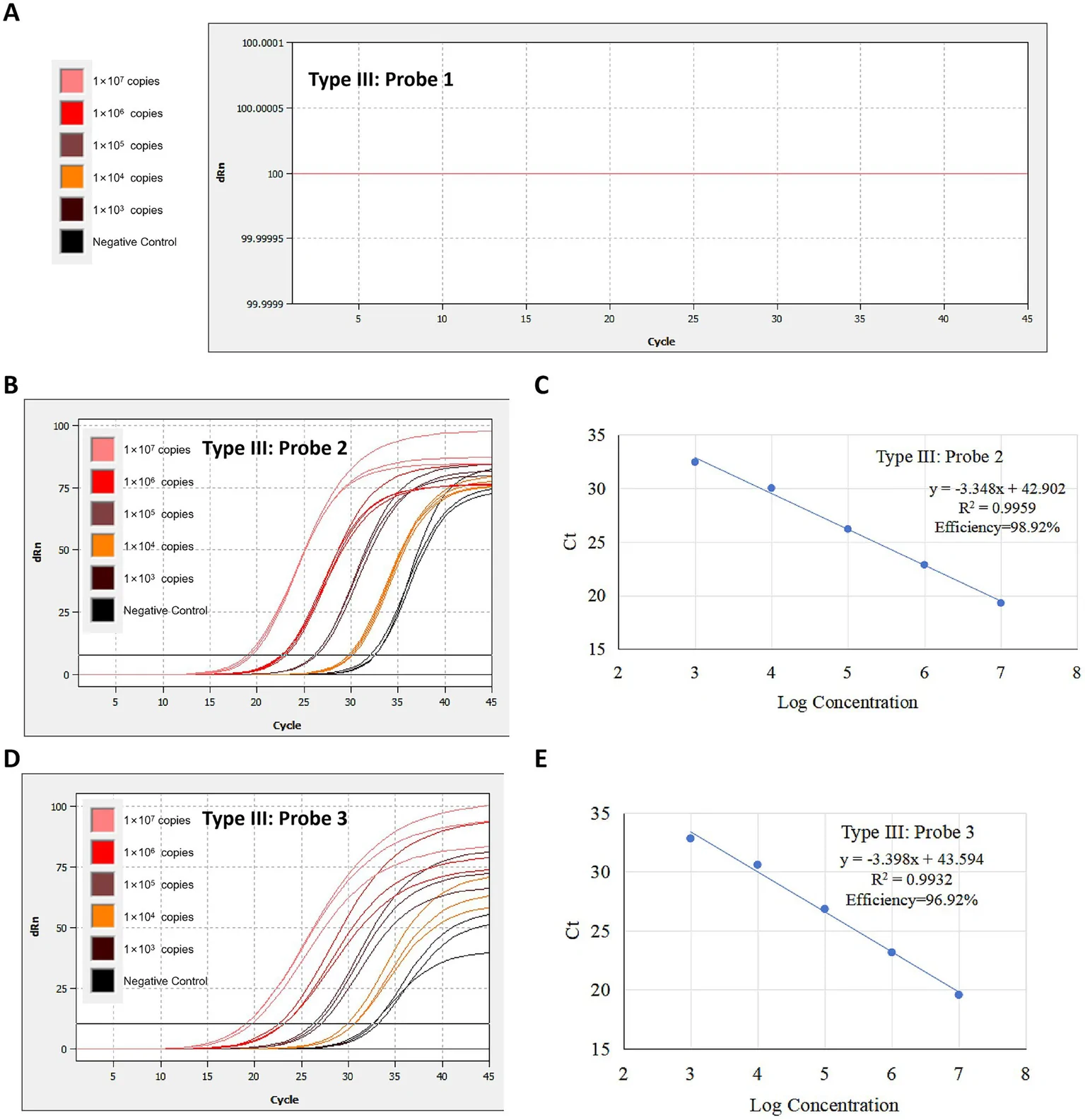
Analytical validation of the TaqMan MGB probe-based assay for detection of the Type III-associated ROP18 pattern. A recombinant plasmid containing the Type III-associated ROP18 pattern sequence was serially diluted from 1 × 10^7^ to 1 × 10^3^ copies/reaction and analyzed using three lineage-associated TaqMan MGB probes. **(A)** Amplification curves generated using Probe 1, showing no detectable amplification signal. **(B)** Amplification curves generated using Probe 2 across serially diluted plasmid standards. **(C)** Standard curve analysis of Probe 2, showing the regression equation, correlation coefficient (*R*^2^), and amplification efficiency. **(D)** Amplification curves generated using Probe 3 across serially diluted plasmid standards. **(E)** Standard curve analysis of Probe 3, showing the regression equation, correlation coefficient (*R*^2^), and amplification efficiency.

As shown in Figures 10A,B,D, no amplification signal was detected for Probe 1 across all tested plasmid concentrations, whereas both Probe 2 and Probe 3 generated specific amplification curves from 1 × 10^3^ to 1 × 10^7^ copies/reaction, consistent with the expected fluorescence recognition pattern of the Type III-associated ROP18 sequence. The standard curves generated from Probe 2 and Probe 3 showed good linearity over the tested concentration range. Probe 2 exhibited a regression equation of y = −3.348x + 42.902, with an R^2^ value of 0.9959 and an amplification efficiency of 98.92% (Figures 10B,C). Probe 3 showed comparable quantitative performance, with a regression equation of y = −3.398x + 43.594, an R^2^ value of 0.9932, and an amplification efficiency of 96.92% (Figures 10D,E). These results demonstrate that the designed TaqMan MGB probes can accurately recognize the Type III-associated ROP18 pattern with reliable amplification performance.

The analytical precision of the Type III-associated ROP18 pattern detection was further evaluated by intra-assay repeatability and inter-assay reproducibility analyses using plasmid standards and CTG genomic DNA. As summarized in Table 13, both Probe 2- and Probe 3-based detection showed low variability across the tested template concentrations. For Probe 2, the intra-assay CV values ranged from 0.42% to 1.40%, while the inter-assay CV values ranged from 0.65% to 2.03%. For Probe 3, the intra-assay and inter-assay CV values ranged from 0.99% to 1.97% and 0.68% to 1.79%, respectively. Detection of CTG genomic DNA also showed good reproducibility, with intra-assay/inter-assay CV values of 1.15%/1.37% for Probe 2 and 1.56%/0.89% for Probe 3, respectively. These findings confirm the stability, repeatability, and reproducibility of the TaqMan MGB probe-based assay for identification of the Type III-associated ROP18 pattern.

**Table 13:** Intra-assay repeatability and inter-assay reproducibility of Probe 2- and Probe 3-based TaqMan MGB assays for detection of the Type III-associated ROP18 pattern.

Sample	Repeatability			Reproducibility		
	Mean Ct	SD	CV%	Mean Ct	SD	CV%
Probe 1						
Copy number of plasmid						
1 × 10^3^	–	–	–	–	–	–
1 × 10^4^	–	–	–	–	–	–
1 × 10^5^	–	–	–	–	–	–
1 × 10^6^	–	–	–	–	–	–
1 × 10^7^	–	–	–	–	–	–
Genomic DNA of CTG	–	–	–	–	–	–
Probe 2						
Copy number of plasmid						
1 × 10^3^	32.45	0.26	0.81	32.21	0.21	0.65
1 × 10^4^	30.02	0.25	0.84	30.10	0.30	1.01
1 × 10^5^	26.19	0.11	0.42	26.41	0.54	2.03
1 × 10^6^	22.86	0.28	1.20	22.73	0.35	1.53
1 × 10^7^	19.29	0.27	1.40	19.33	0.35	1.80
Genomic DNA of CTG	22.24	0.26	1.15	22.31	0.31	1.37
Probe 3						
Copy number of plasmid						
1 × 10^3^	32.84	0.33	0.99	32.68	0.33	1.01
1 × 10^4^	30.60	0.42	1.36	30.53	0.21	0.68
1 × 10^5^	26.85	0.42	1.56	26.78	0.35	1.29
1 × 10^6^	23.16	0.34	1.46	23.21	0.24	1.05
1 × 10^7^	19.57	0.38	1.97	19.56	0.35	1.79
Genomic DNA of CTG	22.53	0.35	1.56	22.69	0.20	0.89

Repeatability was evaluated using three technical replicates within the same experiment, whereas reproducibility was assessed using three independent experiments performed on different days. A Type III lineage-associated *ROP18* plasmid standard was serially diluted from 1 × 10^3^ to 1 × 10^7^ copies/reaction. Genomic DNA extracted from the CTG strain was further evaluated for assay validation. Mean Ct values, standard deviations (SD), and coefficients of variation (CV%) were calculated from replicate measurements. A dash (−) indicates no detectable amplification signal.

## 4 Discussion

In this study, we identified a short conserved sequence region within the 3′ flanking region of the *T. gondii* ROP18 gene containing four informative polymorphic sites that generated distinct lineage-associated sequence patterns among the three major clonal lineages. These ROP18 sequence patterns exhibited strong concordance with phylogenetic clustering of the three major lineages, supporting their potential value as informative molecular markers for lineage differentiation. Based on these characteristic patterns, we further developed two complementary PCR-based genotyping approaches, including HRM-PCR and TaqMan probe-based real-time PCR, enabling rapid and accurate identification of the three major *T. gondii* lineages. Collectively, these findings provide a practical and simplified strategy for molecular classification of *T. gondii* strains and demonstrate the potential of conserved non-coding genomic regions as valuable targets for parasite genotyping.

The selection of the ROP18 3′ flanking region as a molecular marker was based on the potential advantages of non-coding genomic regions for strain differentiation. In microbial genetics, flanking and intergenic regions have been widely used for molecular typing because they are generally subject to lower functional constraints than coding sequences and can accumulate more informative polymorphisms, thereby providing higher discriminatory power for strain differentiation (Glazunova et al., 2005; Li et al., 2009; Bartelli et al., 2013). For example, multispacer sequence typing (MST) of *Coxiella burnetii* relies entirely on intergenic spacer regions due to their increased sequence variability, and similar approaches have been successfully applied in *Candida albicans*, where non-coding regions provide valuable genetic markers for population differentiation (Bartelli et al., 2013). In this study, although the ROP18 3′ flanking region exhibited high sequence conservation among analyzed strains, we identified a short conserved region containing four informative polymorphic sites, whose combinations generated distinct lineage-associated ROP18 patterns.

The ROP18 locus is a well-established virulence-associated region in *T. gondii*, with previous studies mainly focusing on 5′ flanking region or coding sequence variation and expression differences (Khan et al., 2009; Costa et al., 2025; Shwab et al., 2016; Fukumoto et al., 2020; Subekti et al., 2021). In this study, we identified a short conserved sequence region within the ROP18 3′ flanking region containing four informative polymorphic sites, whose combinations generated distinct lineage-associated ROP18 patterns. These lineage-associated ROP18 patterns were not directly equivalent to parasite virulence phenotypes. In our study, TgDogCo17 was identified as carrying the Type I-associated ROP18 pattern. Interestingly, this strain has also been reported to exhibit a highly virulent phenotype (Zhang et al., 2019), suggesting a potential association between the Type I-associated ROP18 pattern and virulence characteristics in certain *T. gondii* genetic backgrounds. However, the presence of Type II- or Type III-associated ROP18 patterns does not necessarily indicate an intermediate- or low-virulence phenotype. Although the Type III-associated ROP18 pattern was identified in TgCatJp5, which has been reported as an avirulent strain (Ihara et al., 2024), the same pattern has also been observed in strains exhibiting different virulence phenotypes. For example, p89 carrying a Type III-associated ROP18 pattern has been reported to display an intermediate virulence phenotype (Khan et al., 2009), whereas CASTELLS, another strain harboring the Type III-associated ROP18 pattern, is considered a highly virulent strain (Khan et al., 2009; Jensen et al., 2015). Similarly, TgCatJpOk4 carrying a Type II-associated ROP18 pattern has also been reported to exhibit high virulence (Taniguchi et al., 2019). The high virulence phenotype of strains carrying a Type III-associated ROP18 pattern may result from additional genetic determinants or alternative virulence pathways beyond the classical ROP18-mediated mechanism (Jensen et al., 2015).

High-resolution melting (HRM) analysis is a rapid and closed-tube genotyping approach that enables differentiation of sequence variations based on differences in DNA melting behavior without the need for post-PCR processing or fluorescent probes (Wittwer et al., 2003). In this study, the identified lineage-associated ROP18 patterns consisted of a short conserved sequence region containing informative polymorphic sites. The successful differentiation of Type I, Type II, and Type III lineage-associated ROP18 patterns using both plasmid standards and representative genomic DNA samples demonstrates the feasibility of HRM-PCR for rapid lineage classification. Compared with conventional multilocus genotyping approaches, HRM-PCR provides a simpler, faster, and cost-effective strategy that is suitable for large-scale molecular epidemiological investigations (Erali and Wittwer, 2010). Previous studies have demonstrated that HRM-based assays can successfully differentiate *T. gondii* genotypes by targeting polymorphic genomic regions, supporting the application of HRM as a practical tool for parasite genotyping (Azimpour-Ardakan et al., 2021; Fotouhi-Ardakani et al., 2026). However, HRM analysis mainly relies on melting profile recognition and does not directly determine the underlying nucleotide variations; therefore, well-characterized reference controls are essential for accurate interpretation. In this study, the use of defined plasmid standards representing the three lineage-associated ROP18 patterns improved assay reliability and enabled consistent differentiation of different lineages. Collectively, these findings indicate that HRM-PCR based on lineage-associated ROP18 patterns represents a practical and efficient approach for preliminary screening and rapid classification of *T. gondii* lineages.

To further improve the specificity and applicability of *T. gondii* lineage differentiation, we developed a TaqMan MGB probe-based real-time PCR assay based on the lineage-associated ROP18 patterns identified in the 3′ flanking region. Compared with HRM-PCR, which relies on differences in melting profiles, TaqMan probe-based assays provide sequence-specific detection through fluorescence signal generation and enable straightforward interpretation of genotyping results (Robert-Gangneux et al., 2017; Kowalczyk et al., 2026). The use of minor groove binder (MGB) probes improves probe stability and sequence differentiation by increasing the melting temperature of short oligonucleotide probes, making them particularly suitable for detecting closely related sequence variants within short amplicons (Kutyavin et al., 2000). In this study, three probes targeting informative polymorphic sites within the ROP18 3′ flanking region were designed, and specific probe-recognition combinations were established for the Type I, Type II, and Type III lineage-associated ROP18 patterns. The assay demonstrated excellent analytical performance, including high amplification efficiency, strong linearity, and good intra- and inter-assay reproducibility, consistent with the recommended evaluation criteria for real-time PCR-based assays (Bustin et al., 2025). Similar TaqMan-based approaches have been successfully applied for *T. gondii* detection and molecular characterization, demonstrating their feasibility for parasite genotyping and epidemiological investigations (Menotti et al., 2010). Although further validation using larger collections of clinical and atypical isolates is required, the developed TaqMan MGB probe-based assay provides a rapid and reliable strategy for differentiation of classical *T. gondii* lineages.

Currently, available genotyping approaches include multilocus PCR-restriction fragment length polymorphism (PCR-RFLP), microsatellite typing, multilocus sequence typing (MLST), and whole-genome sequencing (WGS), each with distinct advantages and limitations (Su et al., 2010; Fernandez-Escobar et al., 2022). PCR-RFLP remains one of the most commonly applied approaches for differentiation of the classical Type I, Type II, and Type III lineages; however, it generally requires multiple genetic markers and several experimental procedures, which may limit its application for rapid and large-scale screening (Fernandez-Escobar et al., 2022). Microsatellite typing and MLST provide higher discriminatory power and are particularly valuable for characterization of atypical strains and complex population structures, but they require analysis of multiple loci and relatively complicated workflows (Fernandez-Escobar et al., 2022). Recent advances in sequencing technologies, including next-generation sequencing and nanopore-based sequencing, have further improved the resolution of *T. gondii* genotyping and enabled comprehensive characterization of parasite genetic diversity (Koutsogiannis and Denny, 2024; Kowalczyk et al., 2026). However, these approaches usually require specialized sequencing platforms, bioinformatic expertise, and relatively higher costs, which may restrict their routine application. In contrast, the strategy developed in this study utilizes a single short conserved sequence region within the ROP18 3′ flanking region containing lineage-associated polymorphic sites, enabling rapid differentiation of the three classical *T. gondii* lineages through HRM-PCR and TaqMan MGB probe-based assays. Compared with conventional multilocus genotyping approaches, this method reduces assay complexity while maintaining clear lineage-specific differentiation. Although this targeted strategy cannot replace sequencing-based approaches for comprehensive population analysis or identification of novel genetic diversity, it provides a practical, rapid, and cost-effective complementary tool for preliminary lineage screening, molecular epidemiological surveillance, and rapid characterization of classical *T. gondii* strains (Koutsogiannis and Denny, 2024; Kowalczyk et al., 2026; Fernandez-Escobar et al., 2022).

Several limitations should be considered in this study. The developed HRM-PCR and TaqMan MGB probe-based assays were validated using representative reference strains (RH, Pru, and CTG) and plasmid standards representing the three classical lineage-associated ROP18 patterns; however, atypical and recombinant genotypes, geographically diverse isolates, blinded unknown samples, and mixed infections were not evaluated. Although the assays showed good analytical performance under the tested conditions, a formal limit of detection (LoD) analysis, comprehensive non-target specificity assessment, clinical or field validation, and direct comparison with established genotyping methods were not performed. Therefore, further validation using expanded strain collections and more complex sample types is required. In addition, plasmid-based evaluation may not fully reflect the complexity of genomic DNA templates. The SRA-based targeted recovery approach may also be affected by sequencing quality, read coverage variation, and potential reference bias. Finally, the identified ROP18 polymorphic signatures should be considered lineage-associated molecular markers rather than direct predictors of virulence. The present assays should be regarded as proof-of-concept approaches for differentiating lineage-associated ROP18 patterns represented by RH, Pru, and CTG, and further functional and genomic studies are required to clarify the biological significance of these polymorphisms.

In conclusion, this study identified a short conserved sequence region within the *T. gondii* ROP18 3′ flanking region containing informative polymorphic sites that generated three distinct lineage-associated ROP18 patterns. Comparative genomic analysis and phylogenetic evaluation demonstrated that these patterns showed strong consistency with the genetic backgrounds of the analyzed strains, supporting their potential as informative molecular signatures for *T. gondii* lineage differentiation. Based on these patterns, we developed two complementary PCR-based genotyping strategies, including HRM-PCR and TaqMan MGB probe-based real-time PCR assays, which enabled rapid, specific, and reproducible identification of different ROP18-associated strain patterns. These findings highlight the potential value of conserved non-coding regions adjacent to virulence-associated genes as informative targets for molecular typing and provide a practical approach for rapid *T. gondii* strain characterization. Although further validation using larger collections of atypical strains and clinical samples is required, the ROP18 pattern-based strategy may serve as a complementary tool to existing multilocus and sequencing-based genotyping approaches for epidemiological surveillance and population genetic studies.

## Data Availability

The original contributions presented in the study are included in the article/Supplementary material, further inquiries can be directed to the corresponding author.

## Footnotes

1
https://toxodb.org

2
https://www.ncbi.nlm.nih.gov/sra

3
https://megasoftware.net/

4
https://www.ncbi.nlm.nih.gov/tools/primer-blast/

5
www.snapgene.com
