# Supplementary material for: Identification of lineage-associated polymorphisms in the ROP18 3′ flanking region and development of molecular assays for differentiation of Toxoplasma gondii lineages
Source: Front Microbiol. 2026 Sep 1;17:1868079. doi: 10.3389/fmicb.2026.1868079 (PMC13574961; doi:10.3389/fmicb.2026.1868079)
Supplement: Supplementary file 1 [file Data_Sheet_1.DOCX]

**Supplementary Table S1. Identification of lineage-associated ROP18 patterns among additional T. gondii strains based on publicly available SRA datasets**.

| No. | Strain name | SRA accession number(s) | Assigned lineage-associated ROP18 pattern |
| --- | --- | --- | --- |
| 1 | TgCatJp5 | DRX496954 | III |
| 2 | BOF | SRX099778 | I |
| 3 | BRCTgH20005 | SRX157499 | III |
| 4 | BRCTgH21016 | SRX099775 | I |
| 5 | CASTELLS | SRX099789 | III |
| 6 | GAB3-2007-GAL-DOM2 | SRX160123 | I |
| 7 | GUY-2003-MEL | SRX160131 | I |
| 8 | TgBoJp1 | DRX496958 | II |
| 9 | TgBoJp2 | DRX496959 | II |
| 10 | TgCatBr15 | SRX099779 | I |
| 11 | TgCatBr25 | SRX160134 | I |
| 12 | TgCatBr26 | SRX099780 | I |
| 13 | TgCatBr3 | SRX160142 | III |
| 14 | TgCatBr64 | SRX160743 | III |
| 15 | TgCatJp1 | DRX496960 | II |
| 16 | TgCatJp2 | DRX496961 | III |
| 17 | TgCatJp3 | DRX496962 | III |
| 18 | TgCatJp6 | DRX496964 | III |
| 19 | TgCatJpGi1TaJ | DRX496956 | III |
| 20 | TgCatJpObi1 | DRX496957 | II |
| 21 | TgCatJpOk4 | DRX140735 | II |
| 22 | TgCatJpTy1k3 | DRX496955 | II |
| 23 | TgCatPRC3 | SRX099797 | III |
| 24 | TgCkBr141 | SRX160124 | I |
| 25 | TgCkCr1 | SRX160807 | I |
| 26 | TgCkCr10 | SRX099784 | I |
| 27 | TgMonkeyJp1 | DRX496966 | II |
| 28 | WH3 | DRX496967 | II |

**Supplementary Figure S1. Agarose gel electrophoresis analysis of PCR amplification targeting lineage-associated ROP18 patterns.
(A)** PCR amplification of synthetic plasmid standards representing the three lineage-associated ROP18 patterns. Human THP-1 and mouse RAW264.7 genomic DNA (10 ng/reaction) were included as non-target DNA controls to evaluate potential non-specific amplification. **(B)** Detection of the corresponding ROP18 pattern-associated sequences in representative *T. gondii* genomic DNA samples. PCR products were visualized by agarose gel electrophoresis.

### **Supplementary **Figure S2. Agarose gel electrophoresis analysis of PCR amplification compatibility of the ROP18 target region in the presence of TaqMan MGB probes.** (A)** PCR amplification of plasmid standards containing Type I-, Type II-, and Type III-associated ROP18 pattern sequences using the corresponding TaqMan MGB probe conditions. **(B)** PCR amplification of representative T. gondii genomic DNA samples using the same probe conditions. RH, PRU, and CTG genomic DNA samples represented Type I-, Type II-, and Type III-associated ROP18 patterns, respectively. Human THP-1 and mouse RAW264.7 genomic DNA (10 ng/reaction) were included as non-target DNA controls. DNA ladders were used as molecular size references. The expected amplification products (~100 bp) were obtained under all compatible probe conditions, indicating that the addition of TaqMan MGB probes did not interfere with PCR amplification of the ROP18 target region.
